# Postnatal expression of cell cycle promoter Fam64a causes heart dysfunction by inhibiting cardiomyocyte differentiation through repression of Klf15

**DOI:** 10.1016/j.isci.2022.104337

**Published:** 2022-04-30

**Authors:** Ken Hashimoto, Aya Kodama, Momoko Ohira, Misaki Kimoto, Reiko Nakagawa, Yuu Usui, Yoshihiro Ujihara, Akira Hanashima, Satoshi Mohri

**Affiliations:** 1 First Department of Physiology, Kawasaki Medical School, Kurashiki 701-0192, Japan; 2 Laboratory for Phyloinformatics, RIKEN Center for Biosystems Dynamics Research (BDR), Kobe 650-0047, Japan; 3 Department of Electrical and Mechanical Engineering, Nagoya Institute of Technology, Nagoya 466-8555, Japan

**Keywords:** Biological sciences, Cell biology, Stem cells research

## Abstract

Introduction of fetal cell cycle genes into damaged adult hearts has emerged as a promising strategy for stimulating proliferation and regeneration of postmitotic adult cardiomyocytes. We have recently identified Fam64a as a fetal-specific cell cycle promoter in cardiomyocytes. Here, we analyzed transgenic mice maintaining cardiomyocyte-specific postnatal expression of Fam64a when endogenous expression was abolished. Despite an enhancement of cardiomyocyte proliferation, these mice showed impaired cardiomyocyte differentiation during postnatal development, resulting in cardiac dysfunction in later life. Mechanistically, Fam64a inhibited cardiomyocyte differentiation by repressing Klf15, leading to the accumulation of undifferentiated cardiomyocytes. In contrast, introduction of Fam64a in differentiated adult wildtype hearts improved functional recovery upon injury with augmented cell cycle and no dedifferentiation in cardiomyocytes. These data demonstrate that Fam64a inhibits cardiomyocyte differentiation during early development, but does not induce de-differentiation in once differentiated cardiomyocytes, illustrating a promising potential of Fam64a as a cell cycle promoter to attain heart regeneration.

## Highlights

• Overexpression of cell cycle promoter Fam64a in cardiomyocytes causes heart failure
• Fam64a inhibits cardiomyocyte differentiation during development by repressing Klf15
• Transient and local induction of Fam64a in adult hearts improves recovery upon injury
• Fam64a activates cardiomyocyte cell cycle without dedifferentiation upon injury

## Graphical abstract

**Figure:**
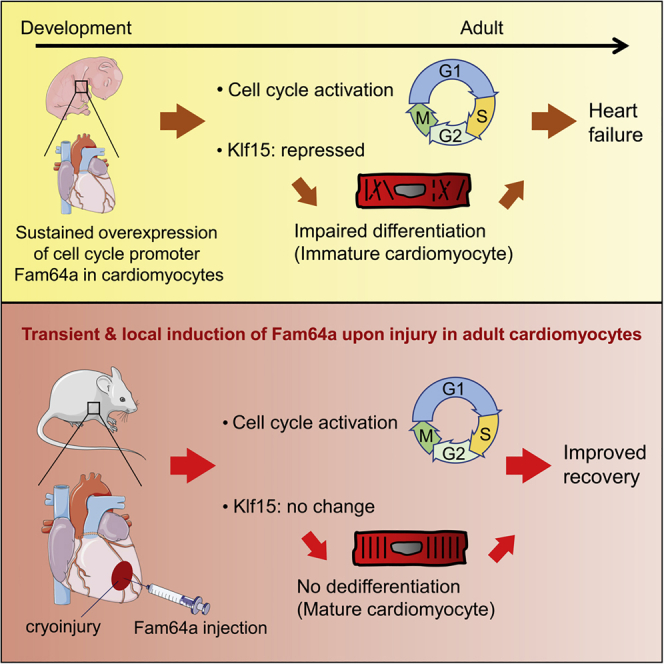

## Introduction

The limited proliferation potential of adult cardiomyocytes (CMs) is a major obstacle hindering regeneration of myocardium lost following injury. Introducing fetal-specific signatures into damaged adult hearts is one of the promising strategies that could stimulate CM proliferation (Pöling et al., 2012). This is because fetal CMs are highly proliferative and reveal a striking regenerative capacity following ablation of up to 60% of CMs (Sturzu et al., 2015). This strategy has recently been addressed with regard to various aspects of the fetal signatures, including cell cycle promoting genes (Mohamed et al., 2018), microRNAs (Borden et al., 2019), epigenetics (Wang et al., 2019), metabolic profiles (Honkoop et al., 2019), and hypoxic environments (Nakada et al., 2017).

We have recently identified family with sequence similarity 64, member A (Fam64a; also known as Pimreg, Cats, or Rcs1) as a fetal-specific cell cycle promoter in CMs (Hashimoto et al., 2017). The strong nuclear expression of Fam64a in fetal CMs was almost completely lost in postnatal CMs from mice (Hashimoto et al., 2017) and sheep (Locatelli et al., 2020). Fam64a knockdown inhibited and its overexpression enhanced fetal CM proliferation *in vitro* (Hashimoto et al., 2017). This proliferation promoting function was also noted in various cancer cell lines (Jiang et al., 2019; Yao et al., 2019). In addition to its proliferation role, Fam64a also enhanced cell migration in several cell lines (Jiang et al., 2019; Yao et al., 2019), and this enhancement was coupled to the epithelial-to-mesenchymal transition (Yao et al., 2019; Zhang et al., 2019), a process that is closely related to cellular dedifferentiation. Fam64a expression was higher in cancer patients with metastasis than in non-metastasis patients (Wei et al., 2019). Fam64a also enhanced stemness features in breast cancer cells (Zhang et al., 2019). These data prompted us to hypothesize the additional role of Fam64a in maintaining immature undifferentiated states in cells by promoting dedifferentiation or inhibiting differentiation. Thus, the aim of this study is (1) to explore the additional function of Fam64a in CMs, and (2) to test how these functions are implicated in cardiac regeneration.

Here, we analyzed transgenic (TG) mice expressing CM-specific Fam64a driven by alpha myosin heavy chain promoter. These mice maintained long-term Fam64a expression after birth when endogenous expression was abolished (Hashimoto et al., 2017). Despite an enhancement of CM proliferation as expected, the TG mice showed impaired CM differentiation during postnatal development, resulting in cardiac dysfunction in later life characterized by increased expression of immature fetal markers and perturbation of the cardiac rhythm. Rhythmic activity of an organism is tightly coupled to cellular differentiation. The circadian clock is absent in undifferentiated cells, such as zygotes and early embryos, and is gradually established during differentiation (Umemura et al., 2017; Yagita et al., 2010). The established rhythmicity is abolished when differentiated cells are reprogrammed to regain pluripotency (Yagita et al., 2010). Thus, the rhythm disturbance and the impaired differentiation observed in Fam64a TG mice could be regulated by the common mechanisms.

We focused on Krüppel-like factor 15 (Klf15) as a candidate molecule responsible for such mechanisms, because this transcription factor is reportedly involved both in cellular differentiation and the establishment of cardiac rhythmicity. Klf15 has been reported to promote differentiation in several cells including skeletal muscle cells (Dmitriev et al., 2011; Wu et al., 2014), adipocytes (Asada et al., 2011; Lee et al., 2016), and podocytes (Mallipattu et al., 2012). Klf15 is also a principal regulator that establishes cardiac rhythmicity (Jeyaraj et al., 2012; Zhang et al., 2015). A deficiency or excess of Klf15 perturbs rhythmic CM electrical activity and increases susceptibility to ventricular arrhythmias (Jeyaraj et al., 2012). It also controls other rhythmic biological processes, such as bile acid synthesis (Han et al., 2015). Klf15 inhibits pathological cardiac remodeling by repressing the process called a fetal gene program, which involves reactivation of immature fetal genes with an enhancement of CM proliferation (Cui et al., 2018; Fisch et al., 2007; Leenders et al., 2010, 2012).

In the present study, we demonstrate that Fam64a transcriptionally inhibits Klf15, thereby impairing CM differentiation during postnatal development, which leads to cardiac dysfunction coupled with rhythm disturbance in adult TG mice, despite an enhancement of CM proliferation. Thus, we propose a previously unknown function of Fam64a in inhibiting CM differentiation through repression of Klf15, in addition to the role as a cell cycle promoter. In contrast, introduction of Fam64a in differentiated adult wildtype (WT) hearts improved functional recovery upon injury with augmentation of the cell cycle and no apparent dedifferentiation in CMs. These data demonstrate that Fam64a inhibits CM differentiation during early development, but does not induce dedifferentiation in once differentiated adult CMs, which would contribute to the functional recovery upon injury, illustrating a promising potential of Fam64a as a cell cycle promoter to attain heart regeneration.

## Results

### Enhanced CM proliferation in CM-specific Fam64a TG mice

**Figure 1:**
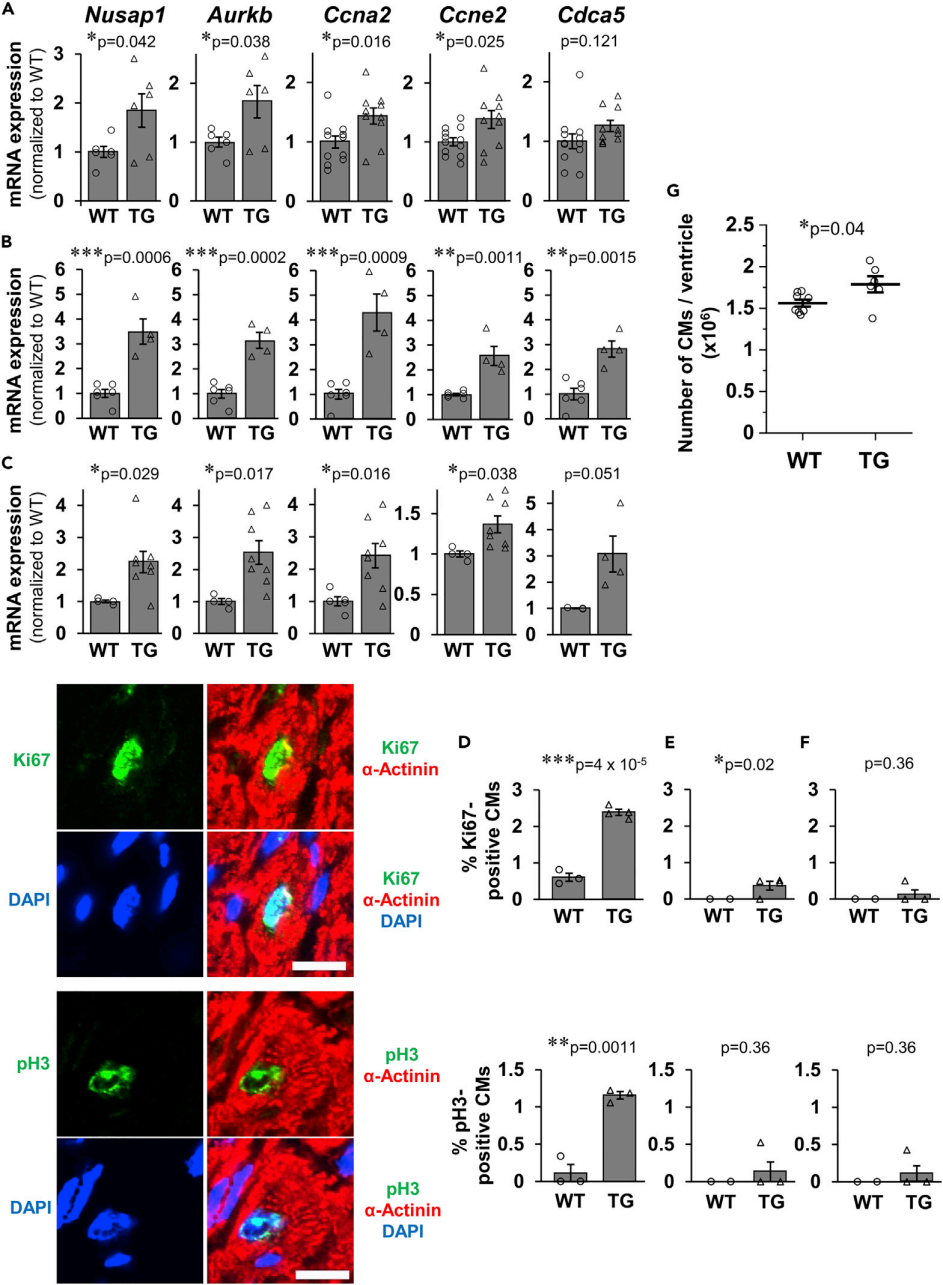
Enhanced CM proliferation in CM-specific Fam64a TG mice (A–C) qPCR analysis of cell cycle promoting genes in WT and TG mice hearts at neonatal (A, P12–P15), adult (B, 6–7 weeks), and aged (C, > 25 weeks) stages. Data were shown as normalized to WT. n = 3–12 mice per group. ∗p < 0.05, ∗∗p < 0.01, ∗∗∗p < 0.001 as compared to WT by Student’s two-tailed unpaired t-test. Error bar = SEM. (D–F) Immunofluorescence for Ki67 and phospho-histone H3 (pH3) observed in sarcomeric α-actinin (as a CM marker) and DAPI in WT and TG mice heart sections at the neonatal (D, P12–P15), adult (E, 6–7 weeks), and aged (F, > 25 weeks) stages. Quantitative analysis for the percentage of Ki67-positive and pH3-positive CMs were shown. n= 3–4 mice per group. ∗p < 0.05, ∗∗p < 0.01, ∗∗∗p < 0.001 as compared to WT by Student’s two-tailed unpaired t-test. Error bar = SEM. Scale bar = 10 μm. (G) Number of CMs per ventricle evaluated by the fixation digestion method in WT and TG mice at 3 weeks. n = 6–8 mice per group. ∗p < 0.05 as compared to WT by Student’s two-tailed unpaired t-test. Error bar = SEM.

We have established CM-specific Fam64a TG mice under the control of alpha myosin heavy chain promoter with a C-terminal FLAG tag (Figures S1A–S1C). Expressed protein was confirmed to localize in the CM nuclei, in the same location as an endogenous protein (Hashimoto et al., 2017) (Figure S1D). We found that the cell cycle promoting genes were slightly, but consistently, upregulated in the hearts of TG mice compared to WT mice at the neonatal (postnatal day, P12–P15), adult (6–7 weeks), and aged (>25 weeks) stages (Figures 1A–1C). We then assessed the CM cell cycle activity by staining for Ki67, a cell cycle marker, and phospho-histone H3 (pH3), a mitosis marker. This revealed that the numbers of both Ki67-positive and pH3-positive CMs were significantly increased at the neonatal stage in TG mice (Figure 1D), but only Ki67-positive CM were increased at the adult stage (Figure 1E), and no increase occurred in either Ki67-positive or pH3-positive CM at the aged stage (Figure 1F). In TG mice, the total CM count per ventricle was increased at 3 weeks (Figure 1G). These data demonstrated an enhanced CM proliferation in TG mice at the neonatal and the juvenile stages, but not at the later stages. These *in vivo* results are in agreement with our previous *in vitro* analyses identifying Fam64a as a CM cell cycle promoter (Hashimoto et al., 2017).

### Fam64a TG mice unexpectedly show cardiac dysfunction with poor survival

**Figure 2:**
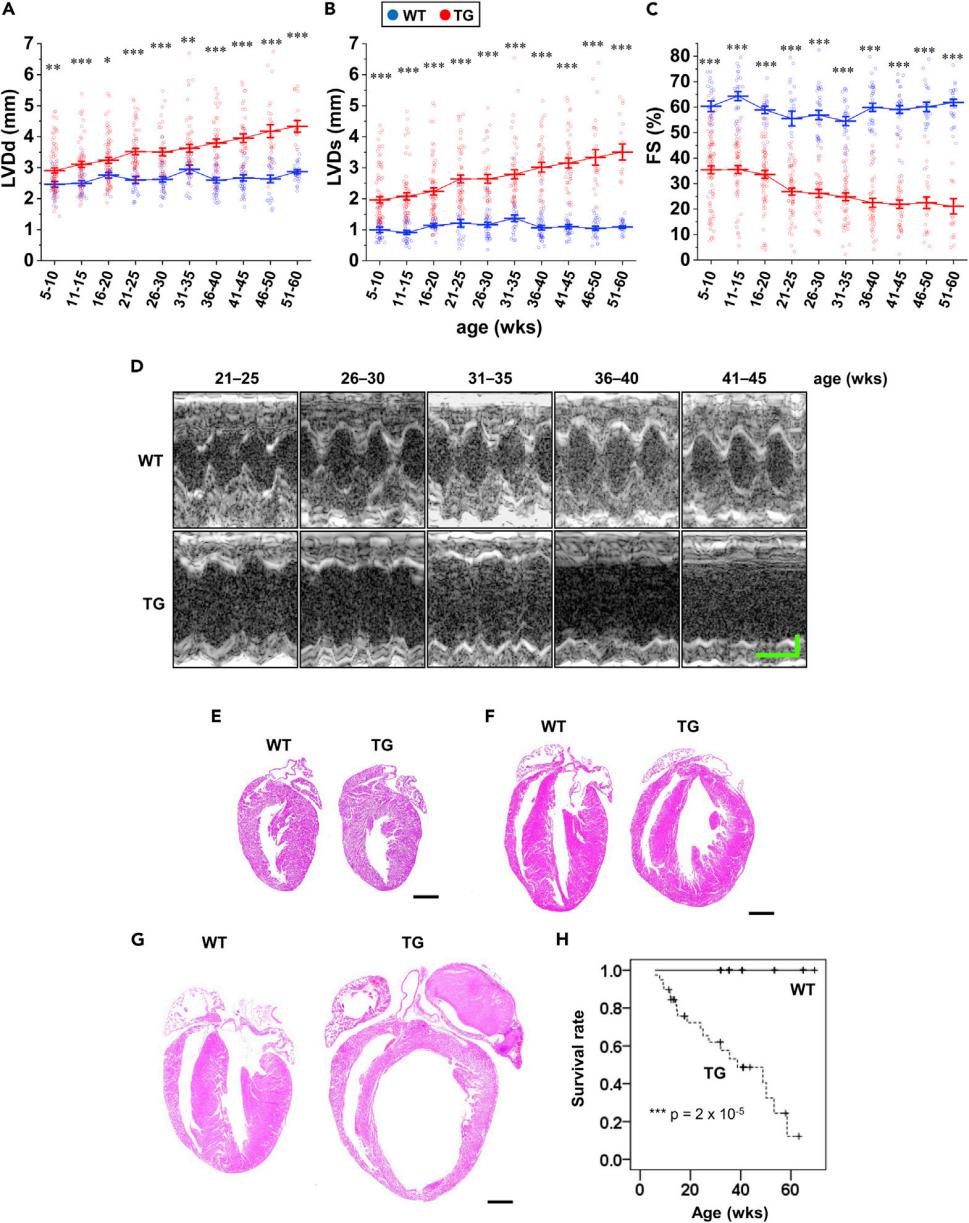
Fam64a TG mice unexpectedly show cardiac dysfunction with poor survival (A–D) Left ventricular internal diameter at end diastole (LVDd, A) and end systole (LVDs, B) were measured in sedated WT (blue) and TG (red) mice from 5 to 60 weeks of age by two-dimensional transthoracic M-mode echocardiography. Fractional shortening (FS, C) was calculated as ([LVDd–LVDs]/LVDd)×100 (%), and was used as an index of cardiac contractile function. Representative tracings were shown in D (Horizontal scale bar = 100 ms, vertical scale bar = 1 mm). In WT mice, no significant change was observed in LVDd, LVDs, and FS over the course of the study, with the only exception of LVDd at 31–35 weeks significantly larger as compared to 5–10 weeks (One-way ANOVA with Tukey’s post hoc test). By contrast in TG mice, LVDd and LVDs were significantly increased, and FS was significantly decreased at 21–25 weeks and afterwards as compared to 5–10 weeks (One-way ANOVA with Tukey’s post hoc test). Consequently, in TG mice, LVDd and LVDs were significantly larger and FS was significantly smaller at all stage as compared to WT mice of the same age (∗p < 0.05, ∗∗p < 0.01, and ∗∗∗p < 0.001 as compared to WT by Student’s two-tailed unpaired t-test.). n > 17 mice per group at each age which partially includes repetitive measurements of the same animal at different age. Error bar = SEM. (E–G) Representative H&E staining for WT and TG mice heart sections at neonatal (E, P12–P15), adult (F, 6–7 weeks), and aged (G, > 25 weeks) stages. Scale bar = 1 mm. (H) Overall survival curves of WT and TG mice were analyzed by Kaplan-Meier method. The vertical line in each plot indicates the censored data. n = 21–39 mice per group. ∗∗∗p < 0.001 as compared to WT by logrank test.

Echocardiography demonstrated that TG mice showed progressive left ventricular dilation both at diastole and systole, leading to a severe decline in cardiac contractile function as estimated by fractional shortening when compared to WT mice (Figures 2A–2D). Histological assessment indicated that although there was no apparent difference at the neonatal stage, chamber dilation and wall thinning in left ventricle was progressively observed in TG mice in the adult and aged stages (Figures 2E–2G). Survival analysis demonstrated a marked drop in survival rate in TG mice (Figure 2H). These data show that Fam64a TG mice develop age-related cardiac dysfunction with poor survival despite their enhanced CM proliferation.

### CM differentiation is impaired during postnatal development, leading to cardiac dysfunction in later life with increased expression of immature fetal markers in Fam64a TG mice

**Figure 3:**
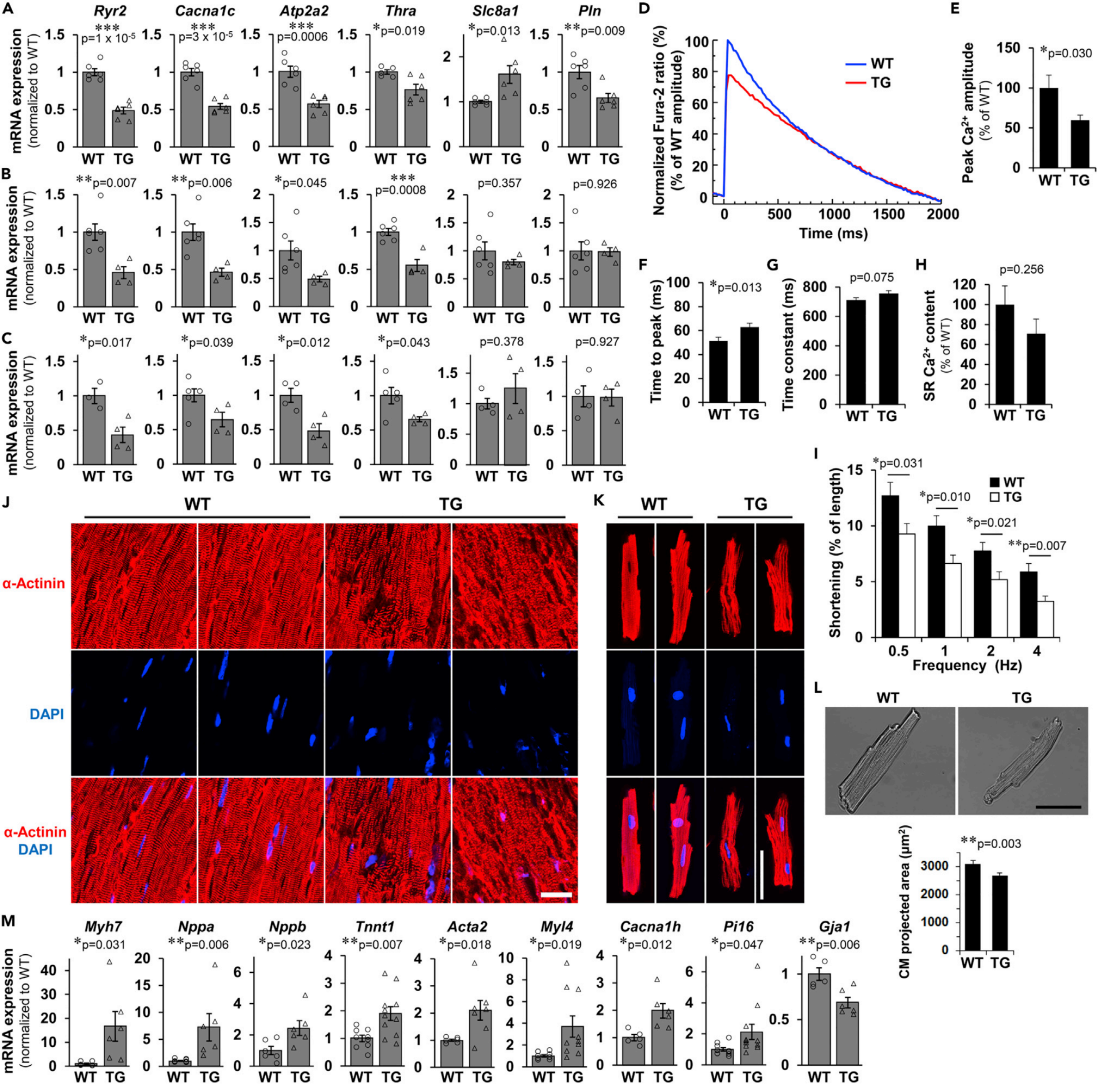
CM differentiation is impaired during postnatal development, leading to cardiac dysfunction in later life with increased expression of immature fetal markers in Fam64a TG mice See also Figures S2–S4. (A–C) qPCR analysis of Ca^2+^ handling genes important for mature differentiated CMs in WT and TG mice hearts at neonatal (A, P12–P15), adult (B, 6–7 weeks), and aged (C, > 25 weeks) stages. Data were shown as normalized to WT. n = 3–6 mice per group. ∗p < 0.05, ∗∗p < 0.01, ∗∗∗p < 0.001 as compared to WT by Student’s two-tailed unpaired t-test. Error bar = SEM. (D–I) Ca^2+^ transients and cell shortening were measured in isolated CMs from WT and TG mice at aged stages (29–32 weeks). Representative Fura-2 ratio tracings of CMs (WT: blue, TG: red) stimulated at 0.5 Hz were shown as normalized to the peak value in WT set at 100% (D). Quantitative analysis for peak Ca^2+^ amplitude (% normalized to WT) (E), time to peak (F), time constant (G), and sarcoplasmic reticulum (SR) Ca^2+^ content (% normalized to WT) (H) were shown. Cell shortening (% of initial cell length) stimulated at indicated frequencies were shown in (I). WT: filled bar, TG: open bar. n = 9–28 CMs from 3 WT mice and 23–50 CMs from 2 TG mice. In (E–H), ∗p < 0.05 as compared to WT by Student’s two-tailed unpaired t-test. In (I), ∗p < 0.05 and ∗∗p < 0.01 as compared to WT under the same stimulating frequency by Student’s two-tailed unpaired t-test. Error bar = SEM. (J–K) Representative immunofluorescence images for sarcomeric α-actinin (red) and DAPI (blue) in longitudinal heart sections (J) and isolated CMs (K) from WT and TG mice at > 25 weeks. In WT mice, highly organized sarcomere structure was observed. In contrast, disorganization of sarcomeres was frequently observed in TG mice. Scale bar = 20 μm (J) and 50 μm (K). (L) Representative images of freshly isolated CMs from WT and TG mice at > 25 weeks aged stages, obtained by differential interference contrast optics. CM cell size was evaluated as a two-dimensional projected area. n = 75 CMs from 3 WT mice and 113 CMs from 2 TG mice. ∗∗p < 0.01 as compared to WT by Student’s two-tailed unpaired t-test. Error bar = SEM. Scale bar = 50 μm. (M) qPCR analysis of immature fetal genes, and a mature gap junction component connexin 43 (*Gja1*) in WT and TG mice hearts. Data were shown as normalized to WT. n = 5–11 mice per group. ∗p < 0.05 and ∗∗p < 0.01 as compared to WT by Student’s two-tailed unpaired t-test. Error bar = SEM.

qPCR analysis demonstrated that Ca^2+^ handling genes that are important for mature differentiated CMs, including *Ryr2*, *Cacna1c*, and *Atp2a2*, were strongly downregulated in TG mice already at neonatal stages (Figure 3A). Thyroid hormone receptor α (*Thra*), a receptor for thyroid hormone T3 that is a strong inducer of postnatal CM differentiation (Karbassi et al., 2020), was also downregulated (Figure 3A). These reductions were similarly seen at adult and aged stages (Figures 3B and 3C). Similar downregulations were observed in primary cultures of isolated CMs overexpressing Fam64a (Figure S2). Moreover, genes encoding several K^+^ channel subunits, which are involved in electrical activity in mature differentiated CMs (Karbassi et al., 2020), were consistently repressed (Figure S3).

The Ca^2+^ transient measurements in isolated CMs from aged mice (29–32 weeks) revealed a reduction in the peak amplitude and a delay in the time to peak, indicating impaired Ca^2+^ mobilization in TG mice as compared to WT mice (Figures 3D–3F). Although not statistically significant, a tendency was observed toward an increased time constant during the decay phase (Figure 3G) and a decreased sarcoplasmic reticulum (SR) Ca^2+^ content (Figure 3H), suggesting impaired Ca^2+^ re-uptake into the SR in TG mice. Cell shortening in response to electrical stimuli was decreased in TG mice at all the frequencies tested, indicating impairment of the CM contractile properties (Figure 3I). Analyses using tissue sections (Figure 3J) and isolated CMs (Figure 3K) revealed disorganization of the sarcomere structures in TG mice. We also found a decreased CM cell size in TG mice, which is recognized as a less differentiated phenotype (Figure 3L).

Meanwhile, the qPCR analysis demonstrated a strong induction of a variety of immature fetal genes in TG mice, including *Myh7*, *Nppa*, *Nppb*, *Tnnt1*, *Acta2*, *Myl4*, *Cacna1h*, and *Pi16* (Chiang et al.,2009; Cui et al., 2018; Ikeda et al., 2019; Kubin et al., 2011; Peng et al., 2017; Taegtmeyer et al., 2010) (Figure 3M). Most of these changes were confirmed at the protein level (Figure S4). Conversely, mRNA level was decreased for connexin 43 (*Gja1*), a primary component of the mature gap junction (Figure 3M).

Collectively, these data show that in Fam64a TG mice, CM differentiation is impaired during postnatal development, leading to cardiac dysfunction in later life with increased expression of immature fetal markers.

### Perturbed cardiac rhythmicity and locomotor activity in Fam64a TG mice

**Figure 4:**
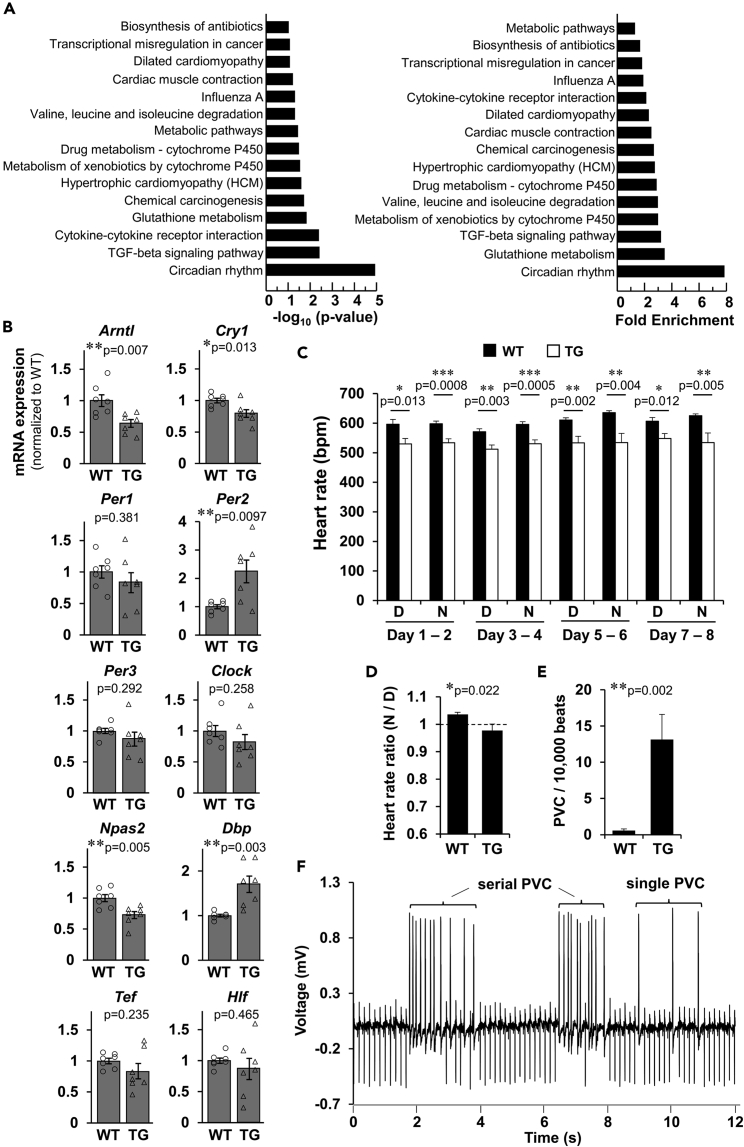
Perturbed cardiac rhythmicity and locomotor activity in Fam64a TG mice See also Figures S5 and S6. (A) Based on RNA-seq data comparing gene expressions in WT vs. TG mice hearts at 6 weeks, functional annotation analysis was performed using DAVID. In this analysis, genes upregulating >2.0 and downregulating <0.5 in TG relative to WT mice were used to identify the differentially regulated gene pathways. The rank order of potency for p value (left) and fold enrichment score (right) were shown. See STAR Methods for details. (B) qPCR analysis of genes involved in circadian rhythm in WT and TG mice hearts. Data were shown as normalized to WT. n = 7 mice per group. Mice at the neonatal (P12), adult (6 weeks), and aged (>25 weeks) stages were mixed. ∗p < 0.05, ∗∗p < 0.01 as compared to WT by Student’s two-tailed unpaired t-test. Error bar = SEM. (C–F) Telemetric ECG measurements using freely moving conscious mice for a total of 8 days in a 12-h light:12-h dark cycle (lights-on at 8 a.m.). C: Averaged heart rate during daytime (D; 8 a.m. to 8 p.m.) and nighttime (N; 8 p.m. to 8 a.m.) in WT (filled bar) and TG (open bar) mice. Data pooled for every 2 days were shown. (D) Nighttime (8 p.m.–8 a.m.)-to-daytime (8 a.m.–8 p.m.) ratio of heart rate in WT and TG mice. Data pooled for 8 days were shown. (E) Frequency of premature ventricular contraction (PVC) per 10,000 beats in WT and TG mice. Data were analyzed for two representative time periods per animal. See STAR Methods for details. (F) Representative ECG tracings in TG mice developing single and serial forms of PVC. In C–F, mice at > 9 weeks were used. Data were analyzed from 6 WT mice and 7 TG mice. ∗p < 0.05, ∗∗p < 0.01, and ∗∗∗p < 0.001 as compared to WT by Student’s two-tailed unpaired t-test. Error bar = SEM.

Surprisingly, RNA-seq analysis of heart samples identified circadian rhythm as the most differentially altered pathway in TG mice, as indicated by an enrichment score far greater than other pathways like cardiac muscle contraction, hypertrophic cardiomyopathy, and dilated cardiomyopathy (Figure 4A, see STAR Methods for details; data have been deposited in DDBJ sequencing read archive, DRA009818). Some of the principal genes relating to circadian rhythm, including *Arntl* (known as Bmal1), *Cry1*, *Per2*, *Npas2*, and *Dbp*, were dysregulated in TG hearts at both the mRNA (Figure 4B) and the protein (Figure S5) levels, although the changes were small. Telemetric measurements using freely moving conscious mice revealed perturbed heart rate regulation in TG mice: (1) Heart rate was consistently low in TG mice, irrespective of daytime or nighttime, throughout the course of measurements of up to 8 days (Figure 4C). (2) Nighttime-to-daytime ratios of heart rate in TG mice were slightly, but significantly, lower than in WT mice, and had values of less than 1, indicating an abnormal daytime (inactive phase)-dominant heart rate regulation (Figure 4D). In addition, TG mice frequently developed premature ventricular contraction, either as a single form or more hazardous serial forms, in sharp contrast to WT mice that displayed virtually no such arrhythmias (Figures 4E and 4F). Decreased expression of connexin 43 (Figure 3M) and K^+^ channel genes (Figure S3) may partially explain these aberrant phenotypes.

Locomotor activity analysis of mice using the infrared motion detector also demonstrated perturbation of rhythmic behavior in TG mice: Whereas the nighttime (active phase)-dominant activity was observed in both WT and TG mice, the activity in TG mice was decreased during nighttime and increased during daytime when compared to WT mice (Figure S6). The abnormal daytime-dominant heart rate regulation (Figure 4D) might be responsible for this phenotype. These data indicate that rhythmic CM electrical activity and locomotor activity were perturbed in Fam64a TG mice.

### Impaired CM differentiation and enhanced CM proliferation observed in TG mice are mediated through transcriptional inhibition of Klf15 by Fam64a

**Figure 5:**
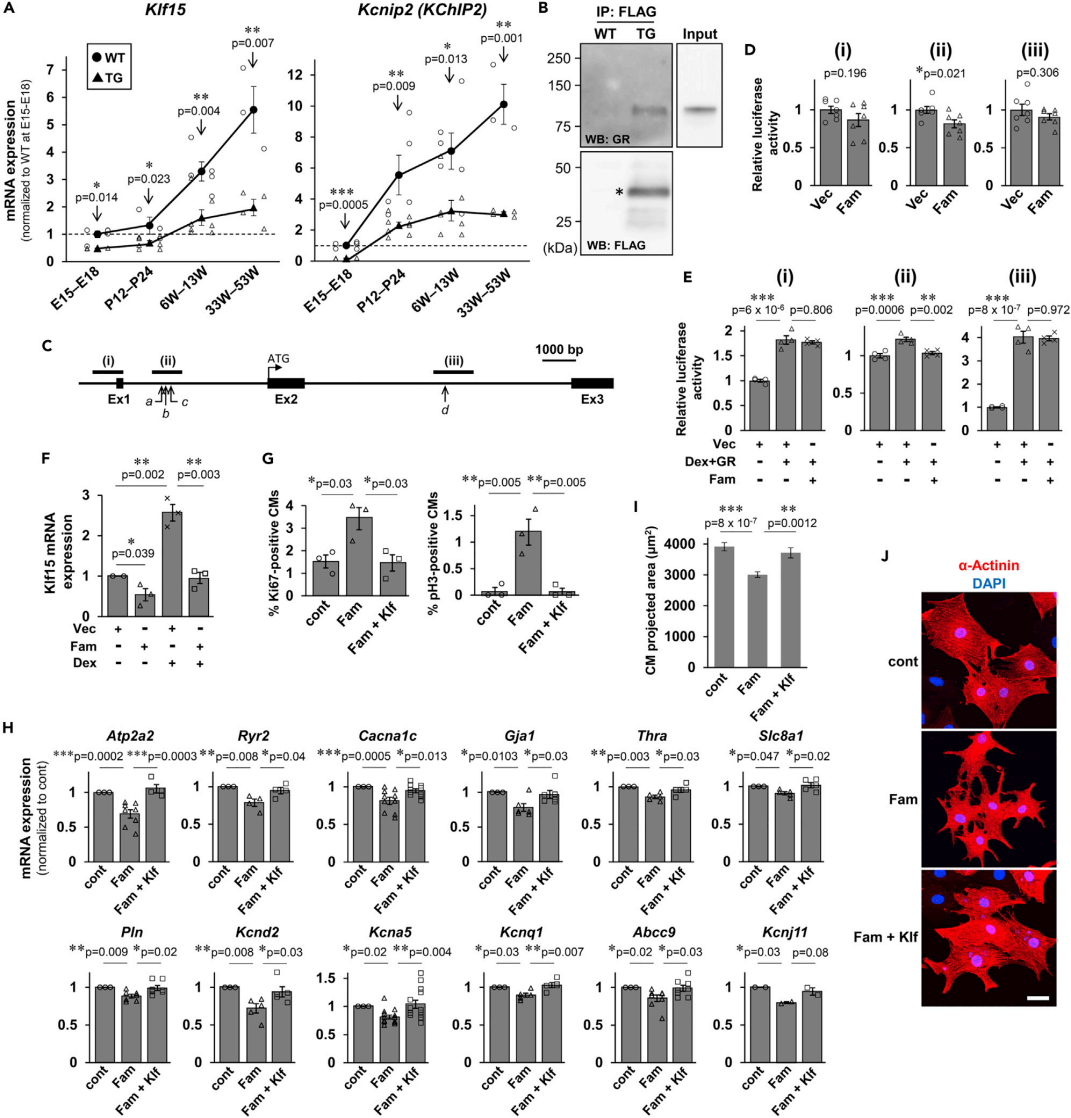
Impaired CM differentiation and enhanced CM proliferation observed in TG mice are mediated through transcriptional inhibition of Klf15 by Fam64a See also Figures S7–S9. (A) qPCR analysis of *Klf15* and *Kcnip2* (KChIP2) at fetal, neonatal, adult, and aged stages from WT (circle) and TG (triangle) mice hearts. Data were shown as normalized to WT at fetal stage set at 1. In WT mice, Klf15 expression was significantly increased at 6–13W and afterward as compared to fetal stage (E15–E18) (One-way ANOVA with Tukey’s post hoc test). Likewise, Kcnip2 expression was significantly increased at P12–P24 and afterward as compared to fetal stage (E15–E18) (One-way ANOVA with Tukey’s post hoc test). By contrast in TG mice, the expressions of both genes were significantly attenuated at all stage as compared to WT mice of the same age (∗p < 0.05, ∗∗p < 0.01, and ∗∗∗p < 0.001 as compared to WT by Student’s two-tailed unpaired t-test.). n = 3–8 mice per group. Error bar = SEM. (B) Immunoprecipitation (IP) against FLAG peptide that was expressed as a C-terminal tag of overexpressing Fam64a protein in TG mice hearts, followed by western blotting (WB) using glucocorticoid receptor (GR) antibody, which detected GR protein in TG, but not in WT mice heart lysates. This indicates that Fam64a interacts with GR in CMs. Western blotting using FLAG antibody correctly detected Fam64a-FLAG fusion protein (∗) in TG, but not in WT mice heart lysates, validating the immunoprecipitation procedure. Three to four mice at > 17 weeks were mixed and used for protein extraction in each genotype. (C) Three reporter constructs on human KLF15 locus were used in luciferase reporter assay. Construct (i) contains common promoter sequence upstream of the first exon. Construct (ii) contains three (marked as *a–c*) of the four GR binding sites previously reported, whilst construct (iii) contains the fourth (marked as *d*). Ex = exon. (D) HEK293T/17 cells were transiently transfected with Fam64a expression vector (Fam) or control empty vector (Vec), and one of the three reporter constructs (ⅰ–ⅲ). The luciferase activity of each reporter construct was normalized to that of the control reporter construct, and was expressed as the activity of Vec set at 1. n = 7 independent experiments. ∗p < 0.05 as compared to Vec by Student’s two-tailed unpaired t-test. Error bar = SEM. (E) HEK293T/17 cells were transiently transfected with Fam64a expression vector (Fam), GR expression vector (GR), or control empty vector (Vec), and one of the three reporter constructs (ⅰ–ⅲ). Cells were treated with dexamethasone (Dex) at 1 μM for 24 h. Luciferase activity of each reporter construct was normalized to that of control reporter construct, and was expressed as the activity of Vec set at 1. n = 4 independent experiments. ∗∗p < 0.01, ∗∗∗p < 0.001 between the indicated groups by One-way ANOVA with Tukey’s post hoc test. Error bar = SEM. (F) Primary CMs were isolated from fetal hearts and transduced with baculovirus expressing Fam64a (Fam) or control empty vector (Vec) in the absence or the presence of dexamethasone (Dex) treatment at 1 μM for 24 h. Total RNA was extracted, reverse-transcribed, and subjected to qPCR analysis for *Klf15*. Data were expressed as *Klf15* mRNA expression of the vector (Vec) group set at 1. n = 3 independent experiments. In each experiment, 5–10 fetal hearts were pooled and used for the isolation of CMs. ∗p < 0.05, and ∗∗p < 0.01 between the indicated groups by Student’s two-tailed unpaired t-test. Error bar = SEM. (G–J) Rescue experiments for Klf15 were performed using primary fetal CMs transduced with baculovirus expressing Fam64a alone (Fam) or both Fam64a and Klf15 (Fam + Klf) to examine whether forced expression of Klf15 restores the phenotypes induced by Fam64a overexpression. In each experiment, 5–10 fetal hearts were pooled and used for the isolation of CMs. (G) The percentage of Ki67-positive (left) and pH3-positive (right) CMs in each condition quantified from immunofluorescence images observed in sarcomeric α-actinin (as a CM marker) and DAPI. n = 3 independent experiments. In each experiment, 100–600 CMs were counted. ∗p < 0.05 and ∗∗p < 0.01 between the indicated groups by One-way ANOVA with Tukey’s post hoc test. Error bar = SEM. (H) qPCR analysis of genes important for mature differentiated CMs in each condition. Data were shown as normalized to controls. n > 3 independent experiments except for *Kcnj11* evaluated with 2 independent experiments. ∗p < 0.05, ∗∗p < 0.01, and ∗∗∗p < 0.001 between the indicated groups by One-way ANOVA with Tukey’s post hoc test. Error bar = SEM. (I) Evaluation of CM cell size as a two-dimensional projected area quantified from immunofluorescence images for sarcomeric α-actinin (as a CM marker) and DAPI (blue). Representative images were shown in J. n = 56, 50, and 32 CMs for control, Fam, and Fam + Klf group, respectively. ∗∗p < 0.01 and ∗∗∗p < 0.001 between the indicated groups by One-way ANOVA with Tukey’s post hoc test. Error bar = SEM. (J) Immunofluorescence for sarcomeric α-actinin (red) and DAPI (blue) in each condition. Scale bar = 30 μm.

Because Fam64a TG mice showed characteristic phenotypes such as the impaired CM differentiation coupled with rhythm disturbance, we focused on Klf15, a key transcription factor that is reportedly involved in these processes (Fisch et al., 2007; Jeyaraj et al., 2012; Leenders et al., 2010, 2012; Zhang et al., 2015). We found that mRNA expression of Klf15 and its downstream target Kv channel-interacting protein 2 (Kcnip2; also known as KChIP2) (Jeyaraj et al., 2012) was strongly upregulated during the course of differentiation in WT hearts but was severely depressed in Fam64a-overexpressing TG mice hearts, suggesting that Fam64a inhibits Klf15 expression at the transcriptional level (Figure 5A). The extent of the inhibition was correlated with the expression level of Fam64a (Figure S7). We conducted a comprehensive search for interacting partners of Fam64a that could mediate the inhibition of Klf15 by immunoprecipitation (Figure S8A), followed by mass spectrometry (see STAR Methods for details; data have been deposited in ProteomeXchange Consortium via jPOST: PXD020570 and JPST000921.). This analysis led us to focus on glucocorticoid receptor (GR) (Figure 5B), because it has previously been shown to bind to the promoter of Klf15 and stimulate its expression (Asada et al., 2011; Lee et al., 2016; Sasse et al., 2013).

Because Fam64a could be a putative transcriptional repressor (Archangelo et al., 2006, 2013), we tested whether Fam64a inhibits GR-mediated transcriptional activation of Klf15 using luciferase reporter assay in HEK293T/17 cells. Three reporter constructs on the human KLF15 locus were used (Figure 5C). Construct (i) contained a common promoter sequence upstream of the first exon. Construct (ii) contained three of the four GR binding sites that were previously reported (Asada et al., 2011; Sasse et al., 2013), whilst construct (iii) contained the fourth. At baseline, in the absence of exogenous induction of GR signaling, all three constructs showed a weak tendency toward a repressed activity because of Fam64a overexpression (Figure 5D). The repression was most strongly and significantly observed in the construct (ii), which contains the majority of the GR binding sites (Figures 5C and 5D). We observed a similar repression in the construct (ii) following exogenous induction of GR signaling by GR overexpression and dexamethasone treatment (Figure 5E), although the repressive effect was weak. We corroborated these findings using primary cultures of isolated CMs to show that Fam64a repressed Klf15 mRNA expression in the absence or presence of exogenous GR induction by dexamethasone (Figure 5F). Dexamethasone-induced activation of Klf15 was completely blocked by Fam64a. These data indicate that Fam64a inhibits Klf15 expression at least in part by GR-mediated transcriptional regulation through action on the previously described GR binding sites.

We next performed rescue experiments *in vitro* to assess whether forced expression of Klf15 restores the phenotypes induced by Fam64a overexpression by using primary cultures of isolated CMs. Overexpression of Fam64a promoted CM cell cycle progression, as shown by increased positivity for Ki67 and pH3 (Figure 5G). This treatment also inhibited CM differentiation, as shown by reduced expression of genes important for mature differentiated CMs (Figure 5H), increased expression of immature fetal genes (Figure S9), a decreased CM cell size (Figure 5I), and frequent appearance of sarcomere disorganization (Figure 5J). These data indicate that the phenotypes induced in Fam64a TG mice were recapitulated in this *in vitro* setting. All of these phenotypes were almost completely restored by concurrent expression of Klf15 (Figures 5G–5J), suggesting that impaired CM differentiation and enhanced CM proliferation observed in TG mice are mediated through transcriptional inhibition of Klf15 by Fam64a. These data also imply that Klf15 could promote CM differentiation and inhibit CM proliferation.

### Introduction of Fam64a in differentiated adult WT hearts improved functional recovery upon injury with augmentation of the cell cycle and no apparent dedifferentiation in CMs

**Figure 6:**
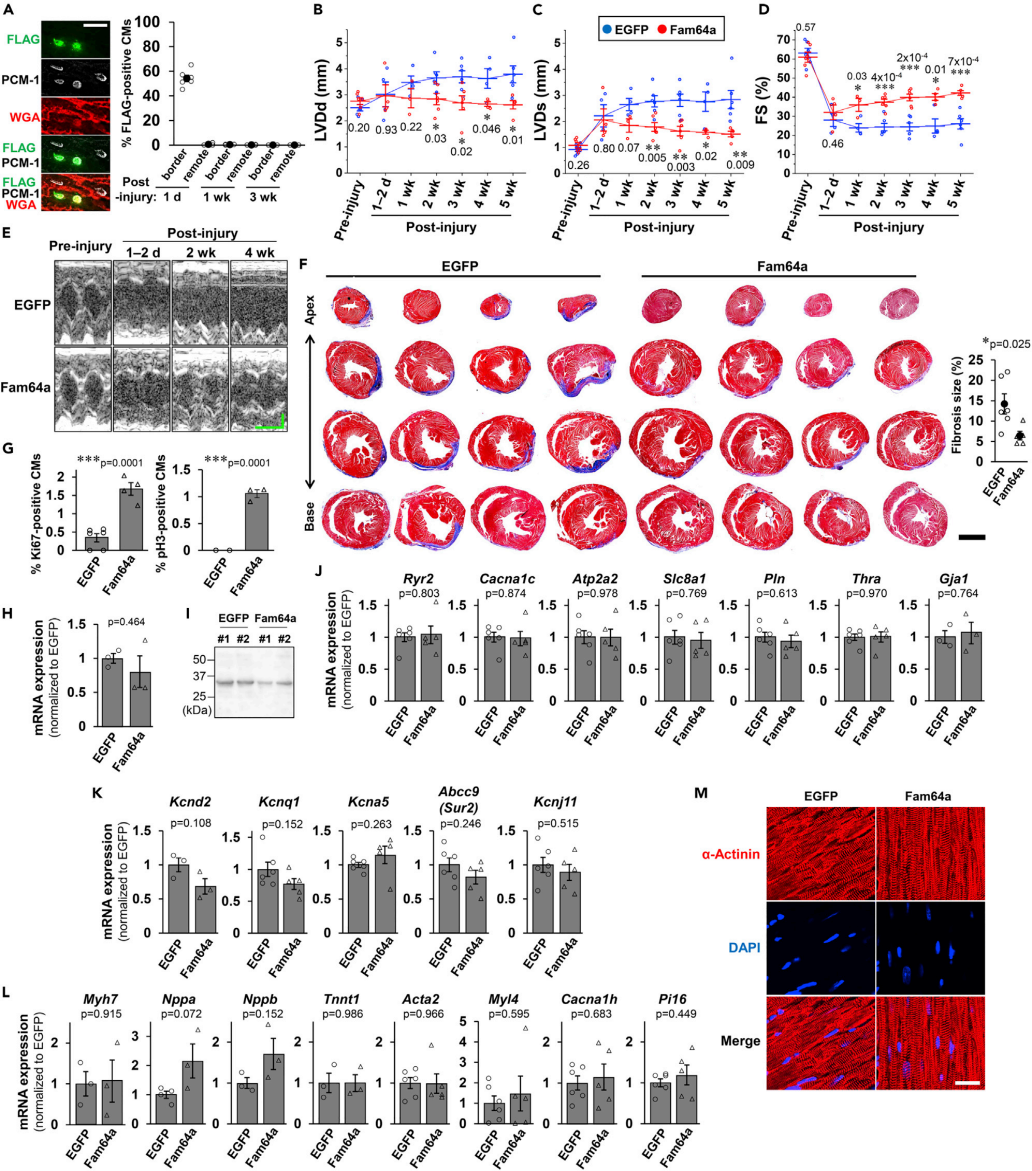
Introduction of Fam64a in differentiated adult WT hearts improved functional recovery upon injury with augmentation of the cell cycle and no apparent dedifferentiation in CMs (A) Time-course analysis for Fam64a-FLAG protein expression in CMs at the injury border and the remote region at 1 day, 1 week, and 3 weeks after injury, evaluated by immunofluorescence with anti-FLAG and anti-PCM-1 (as a CM marker) in heart sections from mice receiving Fam64a-FLAG mRNA. The expressed protein was confirmed to localize in the CM nuclei, in the same location as an endogenous protein. Counterstaining for WGA was performed. Quantitative analysis was shown. n = 3–6 mice per group. Error bar = SEM. Scale bar = 30 μm. (B–E) Cardiac function was evaluated in mice receiving mRNA for EGFP (blue) or Fam64a-FLAG (red) over the course of the experiments. Left ventricular internal diameter at end diastole (LVDd, B) and end systole (LVDs, C) were measured by two-dimensional transthoracic M-mode echocardiography. Fractional shortening (FS, D) was calculated as ([LVDd–LVDs]/LVDd)×100 (%), and was used as an index of cardiac contractile function. Representative tracings were shown in E (Horizontal scale bar = 100 ms, vertical scale bar = 1 mm). n = 4–6 mice in EGFP group and 3–6 mice in Fam64a-FLAG group. ∗ p < 0.05, ∗∗ p < 0.01, ∗∗∗ p < 0.001 as compared to EGFP group at the same stage by Student's two-tailed unpaired t-test. Exact p values were shown on the graph. Error bar = SEM. (F) Representative Masson’s trichrome stainings in heart sections from four mice at 3 weeks after injury in each condition. Quantitative analysis of fibrosis is shown in the right graph. n = 5–6 mice per group. ∗ p < 0.05 as compared to EGFP group by Student's two-tailed unpaired t-test. Error bar = SEM. Scale bar = 2 mm. (G) Quantitative analysis for the percentage of Ki67 and pH3-positive CMs in each condition, evaluated by immunofluorescence for Ki67 and pH3 observed in sarcomeric α-actinin (as a CM marker) and DAPI. n = 3–6 mice per group. ∗∗∗ p < 0.001 as compared to EGFP group by Student's two-tailed unpaired t-test. Error bar = SEM. (H) qPCR analysis for *Klf15* in each condition. Data were shown as normalized to EGFP group. n = 3 mice per group. Error bar = SEM. (I) Western blot analysis for Klf15. Representative blots for two mice in each condition were shown. (J) qPCR analysis of Ca^2+^ handling genes and several genes important for mature differentiated CMs in each condition. Data were shown as normalized to EGFP group. n = 3–6 mice per group. Error bar = SEM. (K) qPCR analysis of genes encoding several K^+^ channel subunits that are important for mature differentiated CMs in each condition. Data were shown as normalized to EGFP group. n = 3–6 mice per group. Error bar = SEM. (L) qPCR analysis of immature fetal genes in each condition. Data were shown as normalized to EGFP group. n = 3–6 mice per group. Error bar = SEM. (M) Representative immunofluorescence images for sarcomeric α-actinin (red) and DAPI (blue) in each condition. Scale bar = 20 μm. In A–M, mice at 13–25 weeks were used.

Based on the findings that cardiac dysfunction in Fam64a TG mice was attributable to impaired CM differentiation early during postnatal development, we next tested whether introduction of Fam64a in differentiated adult WT hearts can circumvent this issue and provide benefits in cardiac regeneration. We used the protocol with direct intramyocardial injection of mRNA encoding Fam64a-FLAG or control EGFP immediately after cryoinjury in WT adult hearts (modified from Kaur and Zangi, 2020; Strungs et al., 2013). The mRNA injection strategy has been reported to achieve a rapid and transient expression of the transgene, which peaks at 24 h and then gradually declines at around ∼10 days. This could avoid the undesired consequences associated with prolonged transgene activation. The expression tends to be spatially confined around the injection/injury site, thereby enabling targeting of the injury site without causing detrimental effects in the remote region. Time-course analysis at the injury border and the remote region at 1 day, 1 week, and 3 weeks after injury revealed expression of the Fam64a-FLAG protein in ∼50% of cardiomyocytes at the border region at 1 day (Figure 6A). As expected, a positive signal was not detected at other timepoints or locations. The estimation by qPCR analysis revealed a 765 ± 251-fold (mean ± SEM, n = 3 mice) increase in the expression of the delivered mRNA in heart samples in comparison to the non-injected controls. Although cardiac contractile function was seriously damaged in both groups immediately after cryoinjury, the mice receiving Fam64a-FLAG mRNA showed progressively improved functional recovery with minimum left ventricular dilation over a follow-up period of 5 weeks in comparison to those receiving control EGFP mRNA (Figures 6B–6E). Histological evaluation by Masson’s trichrome staining revealed less fibrosis in Fam64a-FLAG group at 3 weeks after injury (Figure 6F). The assessment of heart sections containing a cryoinjured area at 5 weeks after injury demonstrated greater cell cycle activity in the Fam64a-FLAG group than the EGFP group, as indicated by increased positivity for Ki67 and pH3 (Figure 6G). Meanwhile, Klf15 expression was slightly decreased both at the mRNA (Figure 6H) and the protein (Figure 6I) levels. Genes important for mature differentiated CMs, which were strongly repressed in TG mice (Figures 3A–C and S3), did not change or only marginally decreased (Figures 6J and 6K). Likewise, immature fetal genes, which were strongly increased in TG mice (Figure 3M), did not change or only marginally increased (Figure 6L). None of the changes in these genes reached statistically significance. Consequently, disorganization of the sarcomere structures, which was frequently observed in TG mice (Figures 3J and 3K), was not observed (Figure 6M). These data demonstrate that Fam64a inhibits CM differentiation during early development, but does not induce dedifferentiation in once differentiated adult CMs, which would contribute to the functional heart recovery upon injury with augmented CM cell cycle.

## Discussion

Current views on the function of Fam64a point to its role as a cell cycle promoter in fetal CMs (Hashimoto et al., 2017) and in various cancer cells (Yamada et al., 2018; Yao et al., 2019). Previous work has led us to hypothesize the additional role of Fam64a in promoting dedifferentiation or inhibiting differentiation to maintain undifferentiated states in cells (Yao et al., 2019; Zhang et al., 2019).

In the TG mice maintaining CM-specific postnatal expression of Fam64a, we saw impaired CM differentiation during postnatal development, resulting in cardiac dysfunction in later life characterized by increased expression of immature fetal markers and perturbation of the cardiac rhythm, despite an enhancement of CM proliferation. Mechanistic analysis and rescue experiments revealed that these phenotypes were mediated through transcriptional inhibition of Klf15 by Fam64a (Figure 5). All of the phenotypes induced by Fam64a overexpression were almost completely restored by concurrent expression of Klf15. A previous study found that Klf15-deficient mice showed perturbed CM rhythmic activity and were susceptible to ventricular arrhythmias, similarly to the effects seen in Fam64a TG mice (Figures 4C–4F), which were considered to reflect suppressed KChIP2 activity (Jeyaraj et al., 2012). KChIP2 is a critical subunit for generating the fast transient outward K^+^ current (I_to,f_) in the early repolarization phase (Kuo et al., 2001), and it augments subsequent Ca^2+^ influx in CMs (Cordeiro et al., 2012; Thomsen et al., 2009). We found severely depressed transcript levels of KChIP2 (Figure 5A) and impaired Ca^2+^ transients (Figures 3D–3H) in TG mice. Suppression of K^+^ channel genes (Figure S3) would also account for the aberrant CM electrical activity. These data suggest that postnatal expression of Fam64a inhibited CM differentiation (Figure 3) through inhibition of Klf15-KChIP2 axis (Figure 5), thereby disrupting CM rhythmic activity (Figure 4) and contributing to cardiac dysfunction (Figure 2), despite an enhancement of CM proliferation (Figure 1). Thus, we propose that Fam64a is not merely a cell cycle promoter; rather, it has an additional role in inhibiting CM differentiation through repression of Klf15. Whether this function of Fam64a is active during fetal development, when endogenous Fam64a is abundantly expressed, needs to be tested.

We demonstrated that GR could, at least in part, mediate the inhibitory effect of Fam64a on Klf15 at the transcriptional level (Figures 5B–5F), although the effect was rather weak. We identified tripartite motif-containing 28 (Trim28) as another interacting partner of Fam64a in CMs (Figure S8B). Trim28 is a known coactivator of GR (Chang et al., 1998). Moreover, the nucleosome remodeling and deacetylase (NuRD) complex, which is implicated in gene repression (Denslow and Wade, 2007), interacts with both Fam64a (Zhao et al., 2008) and Trim28 (Schultz et al., 2001). Therefore, an important remaining challenge is to clarify the mechanism of how protein complexes comprising Fam64a, GR, and Trim28, coupled with the NuRD complex, cooperatively repress Klf15 transcription.

The molecular link between Fam64a and Klf15 found in this study provides a hint at a mechanism by which Fam64a promotes CM proliferation. Multiple lines of evidence point to cell cycle inhibitory action of Klf15 in a variety of non-CM cell types through the regulation of cyclins, cyclin-dependent kinases, cyclin-dependent kinase inhibitors, and DNA synthesis regulators (Hong et al., 2012; Ray and Pollard, 2012; Yoda et al., 2015). Thus, it is possible that Fam64a promotes cell proliferation by relieving this inhibitory action of Klf15 in CMs (Figure 5G).

Recently, Fam64a has been reported to interact with Stat3, and to stimulate its transcriptional activity during colitis-associated carcinogenesis (Xu et al., 2019). Interestingly, like Fam64a, Stat3 induces CM proliferation during cardiac regeneration (Nakao et al., 2020), and has been identified as a factor to acquire undifferentiated pluripotent state in various stem cells (Chen et al., 2015; Nakao et al., 2020). Trim28, an interacting partner of Fam64a (Figure S8B), is recruited to Stat3 target genes to mediate epigenetic activation (Jiang et al., 2018). Thus, it will be intriguing to examine the functional crosstalk between Stat3 pathway and Fam64a-Klf15 axis in CMs. Interestingly, Stat3 transcripts were slightly upregulated in Fam64a TG mice (Figure S10), suggesting an additional layer of regulation of Stat3 activation by Fam64a at the transcriptional level.

In Fam64a TG mice, the impairment of CM differentiation early during development exacerbated cardiac function in later life, despite an enhancement of CM proliferation. In contrast, introduction of Fam64a in differentiated adult WT hearts improved functional recovery upon injury with augmentation of the cell cycle and no apparent dedifferentiation in CMs (Figure 6). These data indicate that Fam64a inhibits CM differentiation during early development, but does not induce dedifferentiation in once differentiated adult CMs. This will make Fam64a a promising candidate as a cell cycle promoter to attain heart regeneration, because several studies have pointed out that excessive CM dedifferentiation evoked by persistent induction of cell cycle stimulants caused cardiac dysfunction (Gabisonia et al., 2019; Ikeda et al., 2019; Kubin et al., 2011), which in some cases could be overcome by an approach of transient induction (D'Uva et al., 2015; Tian et al., 2015). Thus, it is important to optimize an induction protocol for Fam64a, e.g., the intensity and duration that fine-tunes the balance between CM proliferation and dedifferentiation in response to various types of cardiac injury with varying degree of severity. Simultaneous activation of anaphase-promoting complex/cyclosome (APC/C), which targets Fam64a for degradation during each cell cycle (Zhao et al., 2008), will provide another effective means to control the activity of Fam64a.

In summary, this work adds important insights into our understanding of the role of Fam64a. We propose a previously unknown function of Fam64a in inhibiting CM differentiation through repression of Klf15, in addition to the role as a cell cycle promoter. By taking advantage of the feature of Fam64a that does not induce dedifferentiation in adult CMs, future research should aim to identify an optimized induction protocol for Fam64a in injured adult hearts, which will ultimately contribute to development of regenerative therapies of the human heart.

### Limitations of the study

In this study, four limitations should be considered. One was that CMs were generally assumed to require dedifferentiation in order to proliferate; therefore, how CMs activated the cell cycle after cryoinjury, without signs of dedifferentiation such as the changes in Klf15 and related genes, is unclear (Figure 6). However, the concept of CM differentiation/dedifferentiation includes a diverse range of biological processes, such as changes in cell size, T-tubule/sarcomere organization, Ca^2+^ handling, cellular metabolism, and gene expression of mature/immature markers. Of these, the specific condition(s) for promoting dedifferentiation that must be activated to proceed to proliferation is currently uncertain. Therefore, dedifferentiation may have occurred but was not detected by our analyses. Alternatively, some as yet unknown mechanism might allow progression to proliferation by bypassing the dedifferentiation step. A second limitation was that the mechanisms of how the CM cell cycle remained activated at 5 weeks after cryoinjury (Figure 6G) is unclear and needs to be clarified, because the expression of external Fam64a ceased at 1 week after cryoinjury (Figure 6A). A third limitation was that, although enhanced CM proliferation was clearly shown in TG mice at the neonatal and the juvenile stage (Figure 1), the changes in the cell cycle genes were minor, and RNA for the qPCR analysis was extracted from the whole ventricle, which included non-CMs (Figures 1A–1C). Therefore, this limitation needs to be considered in data interpretation. A fourth limitation was that, although the inhibitory effect of Fam64a on Klf15 was clearly demonstrated (Figure 5), the effect was rather weak, especially for the evaluation of reporter repression (Figure 5E). Therefore, the biological significance should be carefully interpreted.

## STAR★Methods

### Key resources table

**Table:** 

REAGENT or RESOURCE	SOURCE	IDENTIFIER
**Antibodies**		

Rabbit monoclonal anti-Ki67 (clone SP6)	Abcam	Cat#ab16667; RRID: AB_302459
Rabbit polyclonal anti-phospho-histone H3 (Ser-10)	Sigma-Aldrich	Cat#06-570; RRID: AB_310177
Rabbit polyclonal anti-PCM-1	Sigma-Aldrich	Cat#HPA023370; RRID: AB_1855072
Mouse monoclonal anti-α-actinin (clone EA-53)	Sigma-Aldrich	Cat#A7811; RRID: AB_476766
Mouse monoclonal anti-FLAG (clone M2)	Sigma-Aldrich	Cat#F1804; RRID: AB_262044
Rabbit polyclonal anti-Klf15	Novus Biologicals	Cat#NBP2-24635
Rabbit monoclonal anti-Trim28 (clone C42G12)	Cell Signaling Technology	Cat#4124; RRID: AB_2209886
Mouse monoclonal anti-GR (clone G-5)	Santa Cruz	Cat#sc-393232; RRID: AB_2687823
Rabbit monoclonal anti-Myh7	ABclonal	Cat#A4963; RRID: AB_2863399
Rabbit polyclonal anti-Nppa	ABclonal	Cat#A14755; RRID: AB_2761631
Rabbit polyclonal anti-Tnnt1	ABclonal	Cat#A10354; RRID: AB_2757899
Rabbit polyclonal anti-Acta2	ABclonal	Cat#A7248; RRID: AB_2721021
Rabbit polyclonal anti-Myl4	ABclonal	Cat#A13249; RRID: AB_2760101
Rabbit polyclonal anti-Arntl	ABclonal	Cat#A17334; RRID: AB_2768451
Rabbit polyclonal anti-Cry1	ABclonal	Cat#A13662; RRID: AB_2760523
Mouse monoclonal anti-Nppb (clone C10)	Abcam	Cat#ab239510
Rabbit polyclonal anti-Dbp	Sigma-Aldrich	Cat#AV31587; RRID: AB_1847497
Rabbit monoclonal anti-Per2	ABclonal	Cat#A5107; RRID: AB_2863447
Rabbit polyclonal anti-Npas2 (clone C1C3)	GeneTex	Cat#GTX105741; RRID: AB_1951013
Rabbit polyclonal anti-Fam64a	This paper	N/A
Goat anti-Rabbit IgG, Alexa Fluor™ 488	Thermo-Fisher	Cat#A11008; RRID: AB_143165
Goat anti-Mouse IgG, Alexa Fluor™ 568	Thermo-Fisher	Cat#A11004; RRID: AB_2534072
Goat anti-Mouse IgG, Alexa Fluor™ 488	Thermo-Fisher	Cat#A11001; RRID: AB_2534069
Goat anti-Rabbit IgG, Alexa Fluor™ 647	Thermo-Fisher	Cat#A21244; RRID: AB_2535812
Anti-Mouse IgG, HRP-Linked Whole Ab Sheep	Cytiva	Cat#NA931; RRID: AB_772210
Anti-Rabbit IgG, HRP-Linked Whole Ab Donkey	Cytiva	Cat#NA934; RRID: AB_772206

**Bacterial and virus strains**		

Baculovirus expressing Fam64a (pFastBac1-VSVG-CMV-Fam64a-WPRE)	This paper	N/A
Baculovirus expressing Klf15 (pFastBac1-VSVG-CMV-Klf15-WPRE)	This paper	N/A

**Chemicals, peptides, and recombinant proteins**		

3× FLAG peptide	Sigma-Aldrich	Cat#F4799
Lipofectamine® 2000	Thermo-Fisher	Cat#11668027
Lipofectamine™ RNAiMAX	Thermo-Fisher	Cat#13778075
Dexamethasone	Sigma-Aldrich	Cat#D4902
*E. coli* Poly (A) polymerase	New England Biolabs	Cat#M0276
CleanCap® Reagent AG	Tri-Link Biotechnologies	Cat#N-7113
N1-Methylpseudo-UTP	Tri-Link Biotechnologies	Cat#N-1081

**Critical commercial assays**		

Mouse on Mouse (M.O.M.™) Basic Kit	Vector laboratories	Cat#BMK-2202
EZview Red Anti-FLAG M2 affinity gel system	Sigma-Aldrich	Cat#F2426
LightSwitch™ luciferase assay system	SwitchGear Genomics	Cat#LS010
HiScribe T7 High Yield RNA Synthesis Kit	New England Biolabs	Cat#E2040S

**Deposited data**		

RNA seq	DDBJ sequencing read archive	Accession number: DRA009818
Proteomics	ProteomeXchange Consortium via jPOST	Accession number: PXD020570 and JPST000921

**Experimental models: Cell lines**		

HEK293T/17 cells	ATCC	ATCC CRL-11268

**Experimental models: Organisms/strains**		

Mouse: Fam64a TG: C57BL/6N	This paper	N/A

**Recombinant DNA**		

Plasmid: alpha myosin heavy chain/puro rex/*neo*	Kita-Matsuo et al. (2009)	Addgene plasmid #21230
Plasmid: LightSwitch™ Promoter Reporter GoClone™ for KLF15	SwitchGear Genomics	Cat#S710100
Plasmid: KLF15 reporter (ii)	This paper	N/A
Plasmid: KLF15 reporter (iii)	This paper	N/A
Plasmid: pGEM-T-HE-T7AGG-Fam64a-FLAG for mRNA synthesis	This paper	N/A
Plasmid: pGEM-T-HE-T7AGG-EGFP for mRNA synthesis	This paper	N/A

**Software and algorithms**		

MASCOT version 2.6	Matrix Science	https://www.matrixscience.com/
Proteome discoverer 2.2	Thermo-Fisher	https://www.thermofisher.com/jp/en/home/industrial/mass-spectrometry/liquid-chromatography-mass-spectrometry-lc-ms/lc-ms-software/multi-omics-data-analysis/proteome-discoverer-software.html
Scaffold (version Scaffold_4.10.0)	Proteome Software Inc.	https://www.proteomesoftware.com/products
cutadapt 1.1		https://cutadapt.readthedocs.org/en/stable/
Trimmomatic 0.32		http://www.usadellab.org/cms/index.php?page=trimmomatic
Tophat 2.0.14		http://ccb.jhu.edu/software/tophat/index.shtml
Cufflinks 2.2.1		http://cole-trapnell-lab.github.io/cufflinks/
DAVID		https://david.ncifcrf.gov/summary.jsp
MetaMorph version 7.8.0.0	Molecular Devices	https://www.moleculardevices.com/products/cellular-imaging-systems/acquisition-and-analysis-software/metamorph-microscopy
Dataquest ART 4.0	Data Sciences International	https://www.datasci.com/products/software/dataquest-art
ImageJ 1.53f51	National Institutes of Health	https://imagej.nih.gov/ij/
SPSS statistics ver. 26	IBM	https://www.ibm.com/products/spss-statistics

### Resource availability

#### Lead contact

Further information and requests for resources and reagents should be directed to and will be fulfilled by the lead contact, Ken Hashimoto (khashimo@med.kawasaki-m.ac.jp).

#### Materials availability

The Fam64a TG mouse line and other materials generated in this study are available upon request.

### Experimental model and subject details

#### Mice

Mice with a C57BL/6N background were housed in a temperature-controlled room under a 12-h light:12-h dark cycle conditions and were fed a standard chow diet and water *ad libitum*. CM-specific Fam64a TG mice were generated as follows: the murine Fam64a sequence with a C terminal FLAG tag was cloned downstream of the alpha myosin heavy chain promoter (Figure S1). The alpha myosin heavy chain/puro rex/*neo* was a gift from Mark Mercola (Addgene plasmid #21230; http://n2t.net/addgene:21230) (Kita-Matsuo et al., 2009). This transgene construct was purified, linearized, and injected into fertilized oocytes from C57BL/6N background mice (Transgenic Inc, Japan). The resulting pups were genotyped by PCR using genomic tail DNA and seven founder lines were established. Among these, two lines expressing a sufficient amount of the transgene both at mRNA and protein levels were selected and used for subsequent experiments (Figure S1). Wildtype (WT) mice with the same background were used for comparison. This study was performed in strict accordance with the recommendations of the Institutional Animal Care and Use Committee at the Kawasaki Medical School. All of the animals were handled according to approved institutional protocols of the Kawasaki Medical School, and every effort was made to minimize suffering. All experiments were performed in accordance with the relevant guidelines and regulations of the Kawasaki Medical School.

### Method details

#### Immunofluorescence

Frozen heart sections embedded in OCT compound (Tissue-Tek; Sakura, UAE) were cut into 8 μm sections with a cryostat (Leica, Germany), permeabilized, blocked with Blocking-One (Nacalai Tesque, Japan), and labeled with primary antibodies, followed by fluorochrome-conjugated secondary antibodies. Counterstaining for DAPI (nuclei), phalloidin (F-actin), and wheat germ agglutinin (WGA; cell membrane) was also performed. Sections were covered with a fluorescence mounting medium (Dako, USA) and examined using an inverted fluorescence microscope (BZ-X710, Keyence, Japan), or a confocal scanning system mounted on a IX81 inverted microscope (FV-1000, Olympus, Japan) (Hashimoto et al., 2018). The primary antibodies used were for Ki67 (clone SP6, Abcam, UK), phospho-histone H3 at Ser-10 (06-570, EMD Millipore, USA), PCM-1 (HPA023370, Sigma-Aldrich), sarcomeric α-actinin (A7811, Sigma-Aldrich), and the FLAG tag (F1804, Sigma-Aldrich). When using mouse-derived antibodies, the Mouse on Mouse (M.O.M.) Basic Kit (Vector, CA, USA) was used. Essentially the same staining protocol was applied for CMs from aged mice isolated with a fixation digestion method (see below). In freshly isolated fetal CMs, fixation was done with 4% paraformaldehyde before permeabilization.

#### Western blotting

Heart tissues were collected from mice, snap frozen in liquid nitrogen, minced, and homogenized using a Kinematica Polytron homogenizer (PT1600E/2500E; Fisher Scientific, USA) in M-PER extraction buffer, CER cytoplasmic extraction buffer, or RIPA buffer (all from Thermo-Fisher, USA) in the presence of a protease inhibitor cocktail (Thermo-Fisher). Lysates were centrifuged, and the supernatants were used for subsequent analysis. After quantifying the protein yield, equal amounts of protein were separated by SDS-PAGE (Mini-PROTEAN TGX; Bio-Rad, USA), transferred onto PVDF membranes (GE Healthcare, USA), blocked with 5% nonfat milk, probed with primary antibodies followed by secondary horseradish peroxidase (HRP)-conjugated IgG (GE Healthcare), and finally visualized by enhanced chemiluminescence (Western Lightning ECL-Pro, PerkinElmer, USA) using a LAS4000mini luminescent image analyzer (GE Healthcare) (Hashimoto et al., 2017). Primary antibodies used were for Klf15 (NBP2-24635, Novus Biologicals, USA), Trim28 (#4124, Cell Signaling Technology, USA), GR (sc-393232, Santa Cruz Biotechnology, USA), Myh7 (A4963, ABclonal, USA), Nppa (A14755, ABclonal), Tnnt1 (A10354, ABclonal), Acta2 (A7248, ABclonal), Myl4 (A13249, ABclonal), Arntl (A17334, ABclonal), Cry1 (A13662, ABclonal), Nppb (ab239510, Abcam), Dbp (AV31587, Sigma-Aldrich), Per2 (A5107, ABclonal), Npas2 (GTX105741, GeneTex), the FLAG tag (F1804, Sigma-Aldrich), and Fam64a. The Fam64a antibody was raised against a synthetic peptide corresponding to residues 154–172 of mouse Fam64a (CRLSGQMGPHAHRRQRLRRE). Full western blot images were provided in Data S1–S5.

#### Immunoprecipitation and mass spectrometry

We identified the interacting partners of Fam64a using immunoprecipitation against the FLAG peptide that was expressed as a C-terminal tag of the overexpressed Fam64a protein in TG mice hearts, followed by mass spectrometry analysis (n = 2 biological replicates). Immunoprecipitates from WT mice hearts were used as a negative control. Heart tissues were freshly isolated from WT and TG mice, minced, and homogenized using a Kinematica Polytron homogenizer (Fisher Scientific) in IP lysis buffer (Thermo-Fisher) or cytoplasmic extraction reagent I & II (Thermo-Fisher) in the presence of a protease inhibitor cocktail (Thermo-Fisher). After centrifugation and protein quantification, the lysates were subjected to immunoprecipitation using the EZview Red Anti-FLAG M2 affinity gel system (F2426, Sigma-Aldrich) according to the manufacturer’s instructions. Elution of the immunoprecipitates was performed with 3× FLAG peptide (F4799, Sigma-Aldrich). The immunoprecipitation procedure was validated by western blotting using FLAG and Fam64a antibodies, which correctly detected the Fam64a-FLAG fusion protein in TG, but not in WT, mouse heart lysates (Figure S8A).

LC-MS/MS ANALYSIS-- In-solution digestion and nano flow-liquid chromatography tandem mass spectrometry were performed (Oya et al., 2019), with some modifications. In brief, the eluted proteins were digested with 10 μg/mL modified trypsin (Sequencing grade, Promega, USA) at 37°C for 16 h. The digested peptides were desalted with in-house made C18 Stage-tips, dried under a vacuum, and dissolved in 2% acetonitrile containing 0.1% trifluoroacetic acid. The peptide mixtures were then fractionated by C18 reverse-phase chromatography (3 μm, ID 0.075× 150 mm, CERI). The peptides were eluted at a flow rate of 300 nL/min with a linear gradient of 5–35% solvent B over 90 min.

DATABASE SEARCHING-- The raw files were searched against the *Mus musculus* dataset (Uniprot Proteome ID UP000000589 2019.06.11 downloaded, 55,197 sequences; 22,986,518 residues) combined with the FLAG-tagged Fam64a sequence and the common Repository of Adventitious Proteins (cRAP, ftp://ftp.thegpm.org/fasta/cRAP) using MASCOT version 2.6 (Matrix Science) via Proteome discoverer 2.2 (Thermo-Fisher), with a false discovery rate (FDR) set at 0.01. Carbamidomethylation of cysteine was set as a fixed modification. Oxidation of methionine and acetylation of protein N-termini were set as variable modifications. The number of missed cleavage sites was set as 2.

CRITERIA FOR PROTEIN IDENTIFICATION-- Scaffold (version Scaffold_4.10.0, Proteome Software Inc., USA) was used to validate the MS/MS-based peptide and protein identifications. Peptide identifications were accepted if they exceeded specific database search engine thresholds. Protein identifications were accepted if they contained at least two identified peptides. Proteins that contained similar peptides and could not be differentiated based on MS/MS analysis alone were grouped to satisfy the principles of parsimony. Proteins sharing significant peptide evidence were grouped into clusters.

In two biologically independent experiments, a total of 440 proteins were detected under the threshold setting in Scaffold software, as follows: protein threshold of 1.0% FDR, peptide threshold of 0.1% FDR, and Min # peptides = 5. Proteins detected only in TG samples, but not in WT samples, were considered as candidate interacting partners of Fam64a, and the interaction of those proteins with Fam64a in heart tissues was subsequently tested by immunoprecipitation and western blotting using specific antibodies. All mass spectrometry data have been deposited in the ProteomeXchange Consortium via jPOST, with the dataset identifiers PXD020570 and JPST000921.

#### Quantitative PCR (qPCR)

Heart tissues were collected from mice, cut into small pieces, and immediately immersed in RNAlater Stabilization Reagent (Qiagen, Germany). The stabilized tissues were homogenized with a Kinematica Polytron homogenizer (Fisher Scientific), and total RNA was isolated using the ISOGEN or ISOGEN-II systems (Nippon Gene, Japan). For cultured CMs, harvested cell pellets were processed similarly to heart tissues but without the use of the homogenizer. After assessing RNA yield and quality using a NanoDrop One spectrophotometer (Thermo-Fisher), the RNA samples were reverse-transcribed with PrimeScrip RT Master Mix (TaKaRa Bio, Japan), and quantitative real-time PCR was performed using TaqMan Fast Advanced Master Mix in a StepOnePlus real-time PCR system (Applied Biosystems, USA). Quantification of each mRNA was carried out with *Actb* or *Ubc* as reference genes, using the ΔΔC_T_ method (Hashimoto et al., 2018).

#### Luciferase reporter assay

Three reporter constructs spanning the promoter region of human KLF15 locus were used (Figure 5C): construct (i):−694/+228, construct (ii): +1066/+1965, and construct (iii): +9444/+10,643, where the number indicates the genomic position relative to the transcription start site. The sequence of the construct (i) was derived from the LightSwitch Promoter Reporter GoClone (SwitchGear Genomics, USA). The expression vectors were a Fam64a expression vector, GR expression vector, or control empty vector (pFastBac1-VSVG-CMV-WPRE; Hashimoto et al., 2017). HEK293T/17 cells (ATCC CRL-11268) were maintained in DMEM with 5% FBS under standard conditions at 37°C with 5% CO_2_. Cells were plated onto 96-well plates coated with fibronectin, and transient transfection of the reporter construct and the expression vector was carried out using Lipofectamine 2000 (Thermo-Fisher) on the following day. The amount of plasmid used per well was 3 ng for each expression vector/50 ng for each reporter construct. The control empty vector was used to equalize the total amount of DNA for each transfection. The expression of the Fam64a protein was confirmed by western blotting. Cells were treated with dexamethasone (Dex) at 1 μM for 24 h. Luciferase activity was measured on the next day using the LightSwitch luciferase assay system (SwitchGear Genomics) as per the manufacturer’s protocol (Hashimoto et al., 2017). The luciferase activity of each reporter construct was normalized to that of the control reporter construct (pLightSwitch_Prom) and was expressed as the activity of the control empty vector set at 1.

#### Histology

Heart tissues were collected from mice, fixed in 4% paraformaldehyde, embedded in paraffin, and vertically sectioned at a thickness of 3 μm. Hematoxylin-eosin (H&E) staining was performed according to standard procedures. Stained sections were observed with a light microscope (BZ-X710, Keyence, Japan).

#### Echocardiography

Two-dimensional transthoracic echocardiography was performed to evaluate cardiac function using an Aplio 300 system with a 14-MHz transducer (Toshiba Medical System, Japan) (Ujihara et al., 2016). M-mode tracings were used to measure the left ventricular internal diameter at end diastole (LVDd) and end systole (LVDs). Fractional shortening (FS) was calculated as ([LVDd–LVDs]/LVDd)×100 (%), and was used as an index of cardiac contractile function. All examinations were performed on conscious mice to prevent anaesthesia-related impairment of cardiac function. In these non-sedated mice, an FS <65% was considered indicative of the impaired cardiac function (Ivandic et al., 2012).

#### Locomotor activity measurement

The locomotor activity of mice was monitored using an infrared motion detector (Actimo-100, Shinfactory, Japan), which consists of a free moving space (30 × 20 cm^2^) with a side wall equipped with photosensors at 2-cm intervals to scan animal movement (Kurokawa et al., 2011). Activity counts accumulated over a 1-h period were measured for a total of 4 days in a 12-h light:12-h dark cycle (lights on at 8 a.m.). Total activity counts during the daytime (8 a.m.–8 p.m.) and nighttime (8 p.m.–8 a.m.) were considered to reflect the locomotor activity in each phase. During the nighttime, we found that the most mice showed characteristic biphasic patterns of locomotor activity, i.e., the first peak during the time period from 8 p.m. to 2 a.m., and the second peak during the time period from 2 a.m. to 8 a.m. (typical example shown in Figure S6A). Thus, the peak activity counts in each phase were used as a measure of the locomotor activity during nighttime.

#### ECG telemetry

Mice were anesthetized with 3% sevoflurane and implanted with a telemetry transmitter device subcutaneously (PhysiolTel HD-X11, Data Sciences International, USA). Two ECG leads were secured at the apex of the heart and the right acromion. The ECG traces of the conscious mice were continuously recorded with a scheduled sampling (10 s every 1 min) using Dataquest ART 4.0 software (Data Sciences International) for a total of 8 days in a 12-h light:12-h dark cycle (lights on at 8 a.m.). The heart rate was calculated by digital tracking of the ECG RR intervals using the Dataquest software, and averaged over 12-h during daytime (8 a.m.–8 p.m.) and nighttime (8 p.m.–8 a.m.). The frequency of premature ventricular contraction was examined over the two representative periods (10 min each, 5000–6000 beats) per animal, which were selected in the middle of the 8-day measurement period (Day 4 and Day 5).

#### Synthesis of modified mRNA

Murine Fam64a-FLAG or EGFP sequence downstream of T7 promoter was cloned into pGEMHE, and was PCR amplified using a primer set (3′-GTAAAACGACGGCCAGT-5' and 3′-CAGGAAACAGCTATGAC-5′). This PCR product was purified using FastGene Gel/PCR Extraction Kit (Nippon Genetics, Japan), and was used as the template for modified mRNA synthesis. *In vitro* transcription was performed using HiScribe T7 High Yield RNA Synthesis Kit (New England Biolabs, USA) with a customized ribonucleoside blend of GTP (1.5 mM), ATP (7.5 mM), CTP (7.5mM), N1-Methylpseudo-UTP (7.5 mM, N-1081, Tri-Link Biotechnologies), and CleanCap® Reagent AG (6 mM, N-7113, Tri-Link Biotechnologies, USA) (Zangi et al., 2017; Kaur and Zangi, 2020). Following the purification of transcribed mRNA using Fast Gene RNA Premium Kit (Nippon Genetics), Poly (A) tailing reaction was performed using *E. coli* Poly (A) polymerase (New England Biolabs), and mRNA was re-purified with the same kit. The size and the integrity of synthesized modified mRNA was checked by agarose gel electrophoresis, and quantity was determined using a NanoDrop One spectrophotometer (Thermo Scientific).

#### Introduction of Fam64a in cryoinjured WT adult hearts using modified mRNA

Cryoinjury experiments were perfomed using protocols adapted from Strungs et al. (2013). WT male mice (13–25 weeks old) were anesthetized with a mixture of 0.3 mg/kg medetomidine, 4.0 mg/kg midazolam, and 5.0 mg/kg butorphanol via subcutaneous injection (Tashiro et al., 2020). Mice were then intubated and mechanically ventilated at 80 breaths/min with a tidal volume of 1000 μL using a rodent ventilator (SN-480-7, Shinano manufacturing, Japan). Hearts were exposed by a left thoracotomy at the fourth intercostal space, the pericardial sac was gently opened by blunt dissection, and a 3-mm-diameter metal cryoprobe, prechilled with liquid nitrogen for 40 s, was applied to the epicardial surface of left ventricular free wall near the apex for 40 s. Cryoinjured area was visually confirmed as a uniform white spot. Immediately after cryoinjury, 20 μg of modified mRNA for Fam64a-FLAG was delivered via direct intramyocardial injection near the cryoinjured spot in a total volume of 100 μL using Lipofectamine RNAiMAX Transfection Reagent (Thermo-Fisher) (modified from Zangi et al., 2017). Modified mRNA for EGFP was used as a negative control. The chest and skin were closed and mice were allowed to recover on a heating pad until normal respiration was obtained. In this procedure, the delivery of modified mRNA at the time of cryoinjury allowed to avoid a second surgery later, which contributed to reduced mortality rate. Echocardiography was performed before and after cryoinjury to assess cardiac function over the course of experiments as described in the aforementioned section. At the end of the experiments (5 weeks after cryoinjury), mice were sacrificed, and the following analyses were performed using heart samples containing a cryoinjured region: Immunofluorescence for Ki67/pH3 along with sarcomeric α-actinin, western blot for Klf15, and qPCR analysis for genes involved in CM dedifferentiation and the FGP. Masson’s trichrome staining for fibrosis evaluation was performed at 3 weeks after cryoinjury.

#### Assessment of fibrosis

Heart tissues were collected from mice at 3 weeks after cryoinjury, fixed in 4% paraformaldehyde, and embedded in paraffin. Transverse sections were cut at 3 μm thickness at an interval of 150 μm through the entire ventricle, from apex to base. The sections were stained with Masson’s trichrome according to standard procedures, and observed with a light microscope (BZ-X710, Keyence, Japan). Fibrosis was quantified using ImageJ 1.53f51 (National Institutes of Health) based on the scar area (blue) and healthy area (red) in the left ventricle.

#### RNA-seq

Heart tissues were collected from mice, cut into small pieces, and immediately immersed in RNAlater Stabilization Reagent (Qiagen). The stabilized tissues were homogenized with a Kinematica Polytron homogenizer (Fisher Scientific), and total RNA was isolated using ISOGEN or ISOGEN-II system (Nippon Gene). After assessing RNA yield and quality using a 2100 Bioanalyzer (Agilent Technologies, USA), RNA-seq libraries were generated using the TruSeq Stranded mRNA Library Prep Kit (Illumina, USA). The quality of the libraries was checked using the 2200 TapeStation (Agilent Technologies). Paired-end sequencing of the libraries was performed on an Illumina Hiseq 2500 platform (Hokkaido System Science, Japan). The obtained data were processed as follows: known adapters and low-quality regions of the reads were trimmed using cutadapt 1.1 (https://cutadapt.readthedocs.org/en/stable/) and Trimmomatic 0.32 (http://www.usadellab.org/cms/index.php?page=trimmomatic), respectively. The reads were mapped to the mouse reference genome (GRCm38, Release 92) using Tophat 2.0.14 (http://ccb.jhu.edu/software/tophat/index.shtml). Gene expression between samples was compared by calculating normalized expression values for each transcript as fragments per kilobase of exon model per million mapped fragments (FPKM) using Cufflinks 2.2.1 (http://cole-trapnell-lab.github.io/cufflinks/). The expression changes in TG vs. WT mice for more than 20 genes were validated by qPCR. Enrichment of genetic associations within KEGG pathways was determined by functional annotation analysis using DAVID (https://david.ncifcrf.gov/summary.jsp). In this analysis, genes upregulated >2.0 and downregulated <0.5 in TG relative to WT mice were used to identify the differentially regulated gene pathways. RNA-seq data have been deposited in DDBJ sequencing read archive (DRA) under the accession number DRA009818.

#### CM isolation from aged mice

Primary CMs were isolated from the ventricles of mice at aged stages (29–32 weeks) (Ujihara et al., 2019). Briefly, the heart was excised and a cannula was inserted into the aorta. Coronary perfusion was initiated with cell-isolation buffer (CIB; 130 mM NaCl, 5.4 mM KCl, 0.5 mM MgCl_2_, 0.33 mM NaH_2_PO_4_, 22 mM glucose, 50 nM/mL bovine insulin, and 25 HEPES-NaOH (pH 7.4)) containing 0.4 mM EGTA. The perfusate was changed to the enzyme solution in CIB containing 0.3 mM CaCl_2_, 1 mg/mL collagenase type II (Worthington Biochemical, USA), 0.06 mg/mL protease (Sigma-Aldrich), and 0.06 mg/mL trypsin (Sigma-Aldrich). The left ventricles were cut into small pieces and further digested in the enzyme solution for 10–15 min at 37°C by gentle agitation. In this enzyme solution, the CaCl_2_ level was increased to 0.7 mM, and 2 mg/mL BSA was supplemented. After centrifugation at 14 × g for 5 min, the pellet was resuspended in CIB containing 1.2 mM CaCl_2_ and 2 mg/mL BSA, and then incubated for 10 min at 37°C. After a further centrifugation, the cells were resuspended in Tyrode’s solution (140 mM NaCl, 5.4 mM KCl, 1.8 mM CaCl_2_, 0.5 mM MgCl_2_, 0.33 mM NaH_2_PO_4_, 11 mM glucose, and5 mM HEPES-NaOH (pH = 7.4)) containing 2 mg/mL BSA.

#### Cell shortening and Ca^2+^ transient measurements

Electrically evoked cell shortening and Ca^2+^ transients were determined in isolated CMs from aged mice stimulated in an electrical field using a two-platinum electrode insert connected to an isolator (SS-104J, Nihon Kohden, Japan) and a bipolar stimulator (SEN-3401, Nihon Kohden) (Katanosaka et al., 2014). The cells were monitored with a CMOS camera (ORCA flash 4.0, Hamamatsu Photonics, Japan) mounted on the side port of an inverted microscope (IX73, Olympus) with a 20× objective lens (UCplanFLN, Olympus). The Ca^2+^ transients were measured by loading isolated CMs with 5 μM Fura-2 AM (Dojindo, Japan) for 30 min (Honda et al., 2018). The Fura-2-loaded cells were alternately excited at 340 and 380 nm using an LED illuminator (pE-340^fura^, CoolLED). The Ca^2+^ content of the sarcoplasmic reticulum (SR) was evaluated by rapidly applying 10 mM caffeine and measuring the resulting Ca^2+^ transients in isolated CMs. Data were analyzed using MetaMorph version 7.8.0.0 software (Molecular Devices, USA).

#### CM isolation from fetal mice

Primary CMs were isolated from the ventricles of fetal mice at embryonic day E16–E18 (Hashimoto et al., 2018). Briefly, pregnant mice were euthanized with Sevofrane, and fetal heart ventricles were rapidly excised, cut into small pieces, and digested three times with 0.06% trypsin in PBS for 10 min at 37°C. After a 20-min culture to exclude non-CMs, the CMs were plated onto fibronectin-coated culture vessels in DMEM with 10% FBS and cultured under standard conditions at 37°C with 5% CO_2_. In each isolation procedure, 5–10 fetal hearts were pooled and used for the isolation.

#### Fixation digestion method for counting total CMs

The total number of CMs in the ventricle was evaluated with a fixation digestion method (slightly modified from Liu et al., 2021). The heart was excised from mice and washed with cardioplegia solution containing 25 μM KCl. Ventricles were cut into 0.5–1 mm tissue blocks, fixed with 4% paraformaldehyde for 100 min, washed 3 times with PBS, and digested with the enzyme solution containing 3.6 mg/mL collagenase B (11-088-807-001, Sigma-Aldrich) and 4.8 mg/mL collagenase D (11-088-858-001, Sigma-Aldrich) for 24 h at 37°C by gentle agitation. The digested cells were collected, and the rod-shaped CMs were counted with a hemocytometer. The undigested tissues were digested with a new enzyme solution for an additional 24 h. These procedures were repeated until all tissues were digested.

#### Baculovirus-mediated protein expression

We have previously established a baculovirus-mediated protein expression system in fetal CMs (Hashimoto et al., 2017, 2018). A robust expression of target protein in fetal CMs was confirmed by western blot and fluorescence imaging of GFP, which was fused to the target sequence. In this study, a baculovirus expressing mouse Fam64a (NM_144,526.3) or mouse Klf15 (NM_023,184.4) was used. Approximately 300- and 150-fold induction for Fam64a and Klf15, respectively, was observed by qPCR. Baculovirus was produced in Sf9 cells, as per the manufacturer’s instructions (Thermo-Fisher). For transduction to CMs, virus was added to the cells in DMEM without serum. After 7 h, the cells were treated with BacMam enhancer (Invitrogen, USA) for an additional 2 h, according to the manufacturer’s protocol, to increase the transduction efficiency. The medium was then replaced with DMEM containing 10% FBS.

### Quantification and statistical analysis

All data were expressed as mean plus or minus standard error of the mean (SEM). For comparisons between two groups, Student’s two-tailed unpaired t-test was performed using Microsoft Excel 2019 MSO (16.0.10358.20061) to determine statistical significance. For comparisons among multiple groups, one-way analysis of variance (ANOVA) was performed with Tukey’s post hoc test using SPSS statistics ver. 26 (IBM, USA). Kaplan-Meier analysis was performed using SPSS statistics ver. 26 (IBM) to estimate the survival curve of WT and TG mice, and between-group differences were analyzed with the logrank test. p< 0.05 was considered statistically significant. Significance levels were indicated as follows: p < 0.05, ‘∗’ p < 0.01, ‘∗∗’ p < 0.001‘∗∗∗’. Additional statistical information, including sample sizes and p values for each experiment, is detailed in the figure legends.

## Data Availability

• RNA-seq data have been deposited at DDBJ sequencing read archive (DRA009818), and are publicly available as of the date of publication. Accession numbers are listed in the key resources table. • Proteomics data have been deposited at ProteomeXchange Consortium via jPOST (PXD020570 and JPST000921), and are publicly available as of the date of publication. Accession numbers are listed in the key resources table. • This paper does not report original code. • Any additional information required to reanalyze the data reported in this paper is available from the lead contact upon request.
