# Supplementary material for: Postnatal expression of cell cycle promoter Fam64a causes heart dysfunction by inhibiting cardiomyocyte differentiation through repression of Klf15
Source: iScience. 2022 Apr 30;25(5):104337. doi: 10.1016/j.isci.2022.104337 (PMC9118685; doi:10.1016/j.isci.2022.104337)
Supplement: Document S1. Figures S1–S10 and Data S1–S5 [file mmc1.pdf]

iScience, Volume *25*

Supplemental information

Postnatal expression of cell cycle promoter Fam64a causes heart dysfunction by inhibiting cardiomyocyte differentiation through repression of Klf15

Ken Hashimoto, Aya Kodama, Momoko Ohira, Misaki Kimoto, Reiko Nakagawa, Yuu Usui, Yoshihiro Ujihara, Akira Hanashima, and Satoshi Mohri

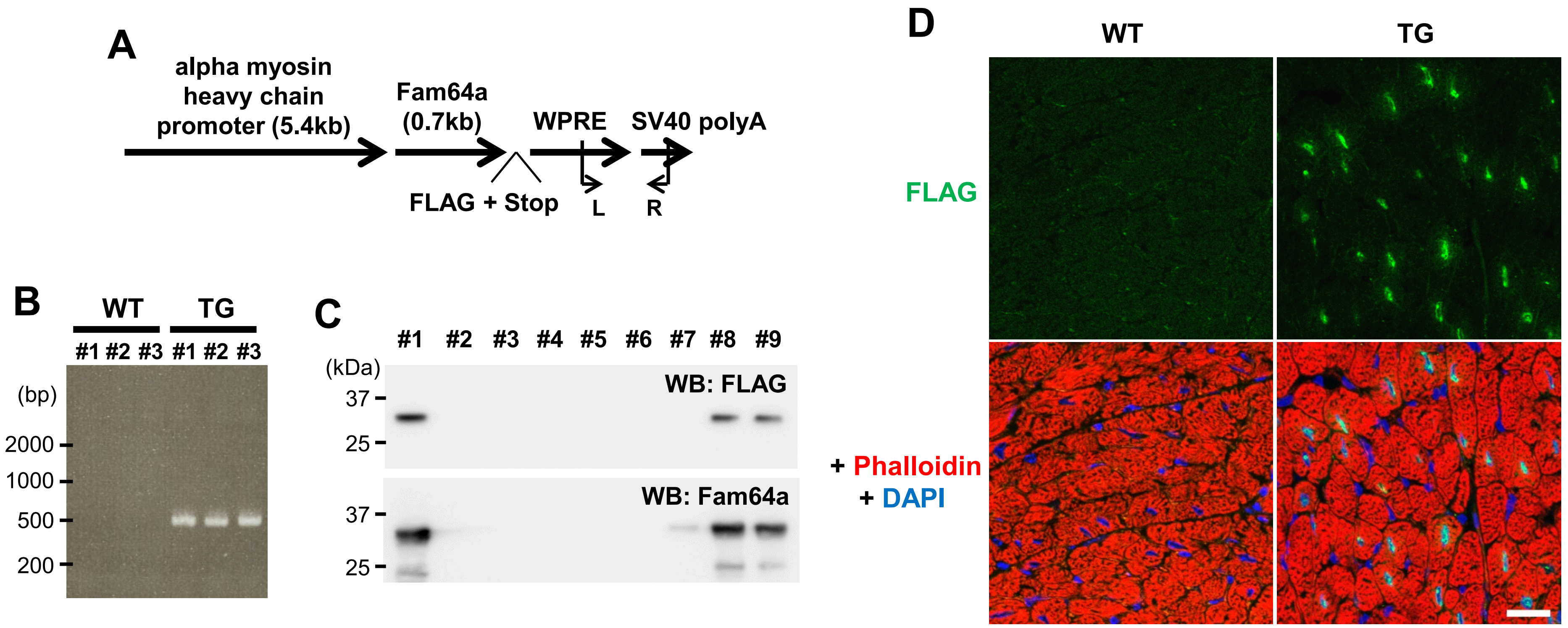

Figure S1. Generation of cardiomyocyte-specific Fam64a TG mice, related to STAR Methods

A. The transgene construct containing murine Fam64a sequence with a C terminal FLAG tag cloned downstream of the alpha myosin heavy chain promoter. WPRE; Woodchuck hepatitis virus Posttranscriptional Regulatory Element for stabilizing transcribed mRNA. **B.** Representative genotyping results using genomic tail DNA from 3 WT and 3 TG mice. Positions for left (L) and right (R) primers were marked in A. **C.** Representative western blots (WB) of heart homogenates from 9 mice at F1 generation (6 wks of age) derived from 7 founder TG lines using anti-FLAG or anti-Fam64a antibody, both of which detect overexpressing Fam64a-FLAG fusion protein. Based on the amount of the protein detected, we classified 7 founder lines into 3 categories; TG-strong (2 lines, for example #1, #8, and #9), TG-medium (2 lines, for example #7), and TG-weak (3 lines, for example #2, #4, and #5). Descendants of TG-strong lines (randomly mixed from the 2 lines) were used for subsequent experiments. The expression level of the transgene was well correlated with the phenotypes of TG mice (see Figure S7). **D.** Immunofluorescence for heart tissue sections from WT and TG mice at 6 wks using anti-FLAG antibody that detects overexpressing Fam64a-FLAG fusion protein. Counterstaining for phalloidin and DAPI was performed. Expressed protein was confirmed to localize in the CM nuclei, in the same location as an endogenous protein. Scale bar = 20 µm.

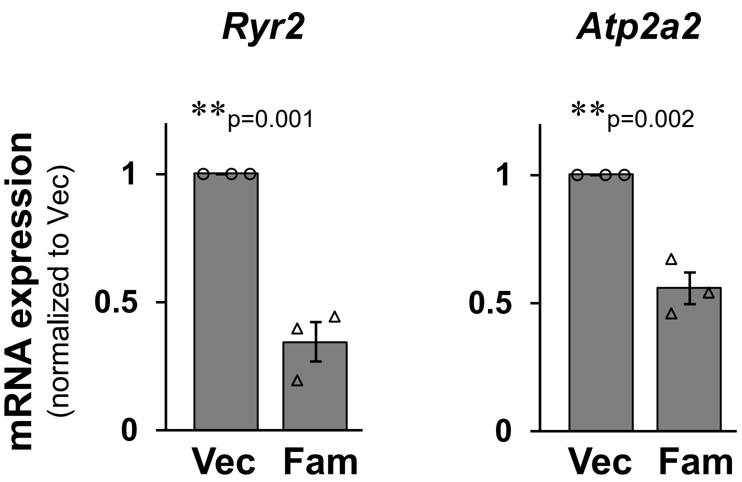

Figure S2. Ca^{2+} handling genes important for mature differentiated CMs were downregulated by Fam64a overexpression in isolated CMs, related to Figure 3

Primary CMs were isolated from fetal hearts and transduced with baculovirus expressing Fam64a (Fam) or control empty vector (Vec). Total RNA was extracted, reverse-transcribed, and subjected to qPCR analysis for *Ryr2* and *Atp2a2*. Data were expressed as mRNA expression in the vector group set at 1. n = 3 independent experiments. In each experiment, 5–10 fetal hearts were pooled and used for the isolation of CMs. ** $p < 0.01$ as compared to Vec by Student's two-tailed unpaired t-test. Error bar = SEM.

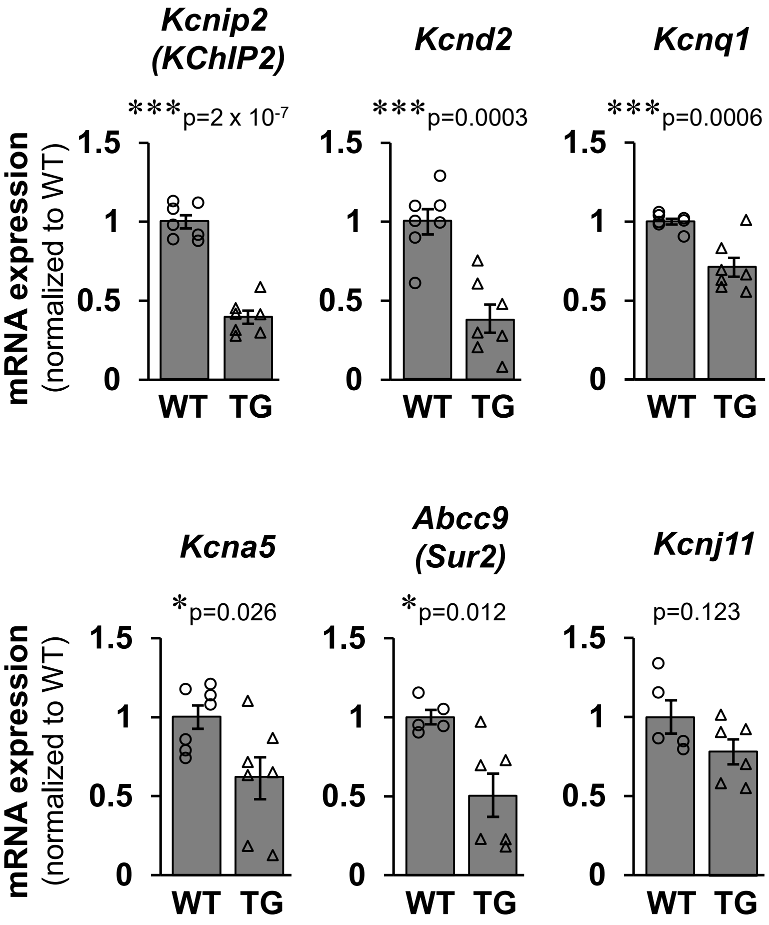

Figure S3. K^+ channel genes important for mature differentiated CMs were consistently repressed in Fam64a TG mice, related to Figure 3

qPCR analysis of genes encoding several K^+ channel subunits in WT and TG mice hearts. Data were shown as normalized to WT. n = 5–7 mice per group. * $p < 0.05$, *** $p < 0.001$ as compared to WT by Student's two-tailed unpaired t-test. Error bar = SEM.

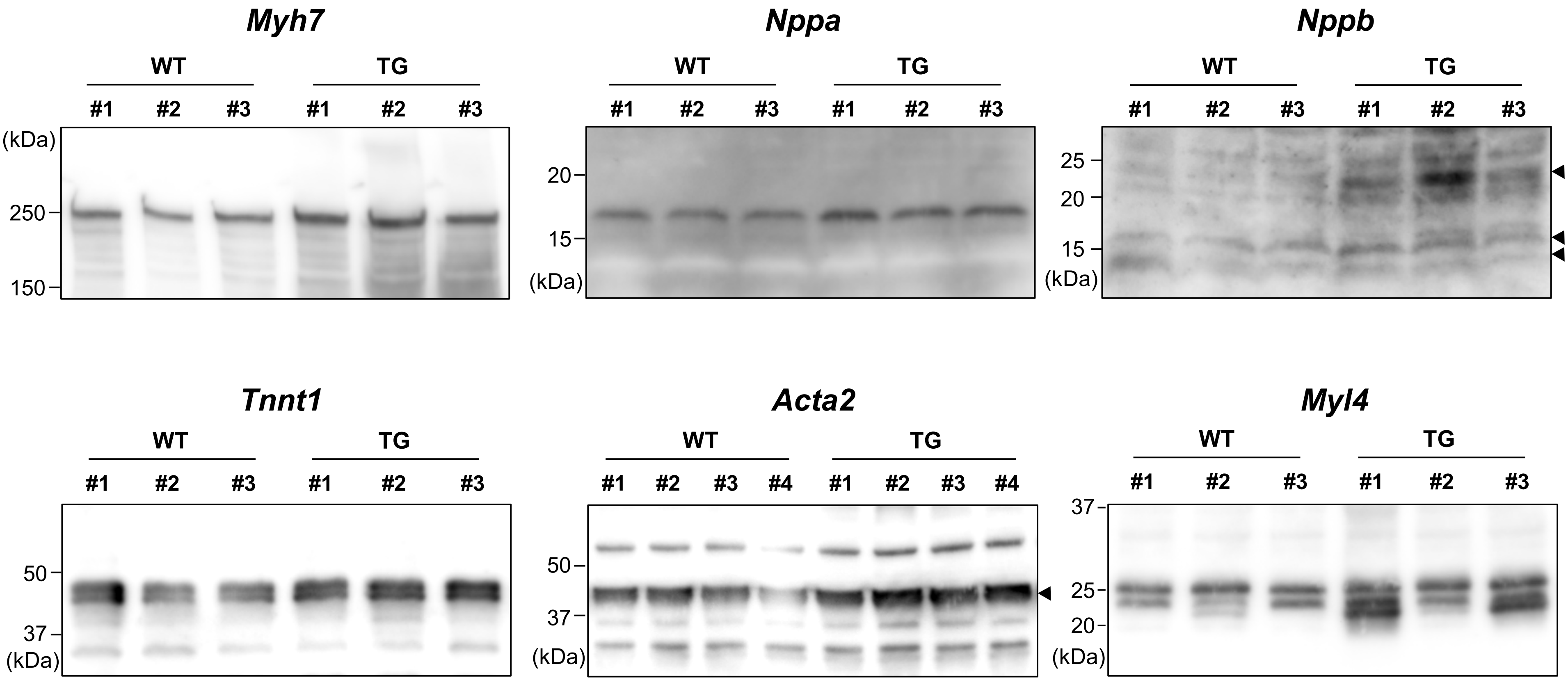

Figure S4. Western blot analysis for immature fetal genes, related to Figure 3
Representative blots of heart homogenates from 3 mice (4 mice for *Acta2*) at 5–7 wks in each genotype.

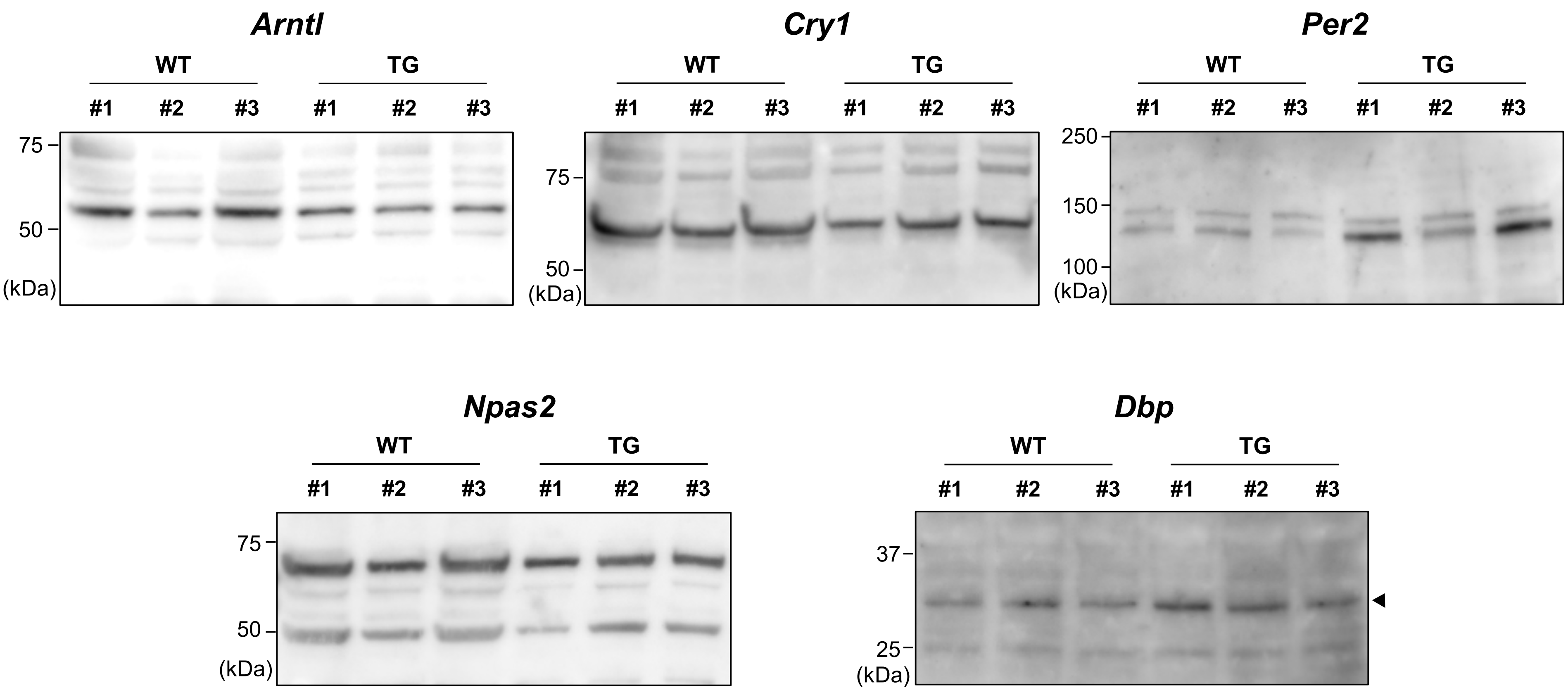

Figure S5. Western blot analysis for circadian rhythm genes, related to Figure 4
Representative blots of heart homogenates from 3 mice at 5–7 wks in each genotype.

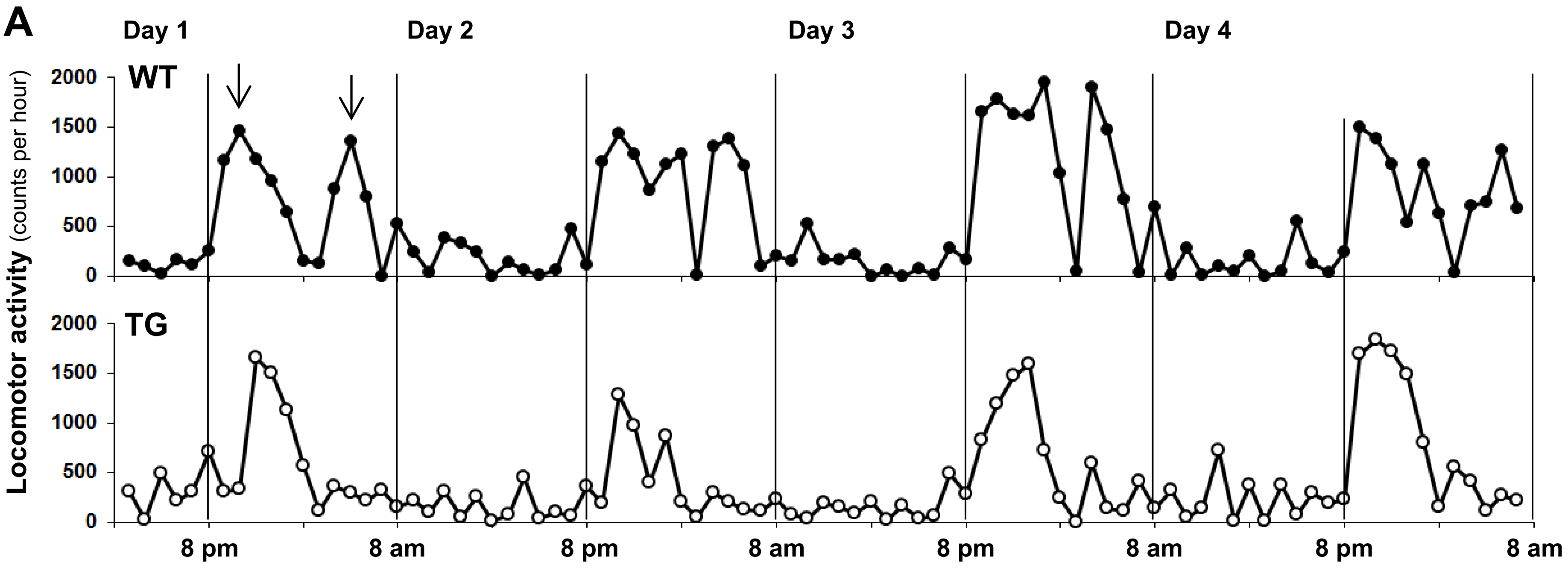

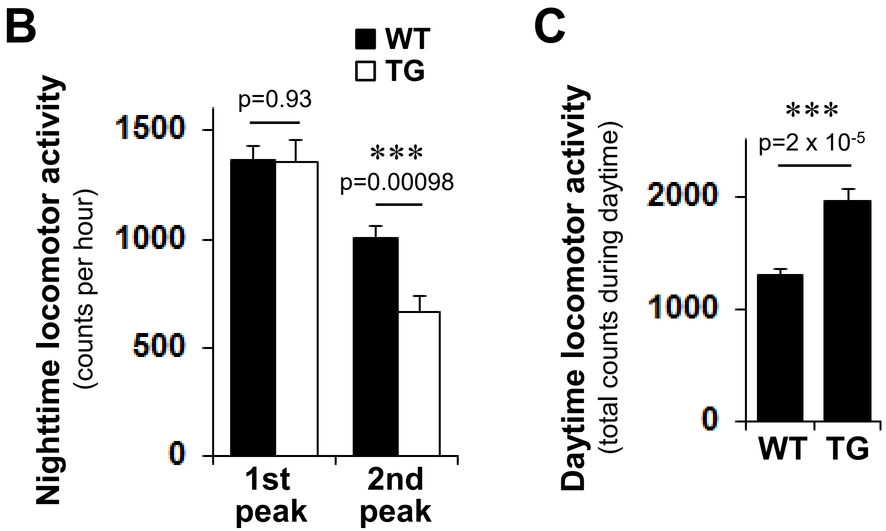

Figure S6. Perturbed locomotor activity in Fam64a TG mice, related to Figure 4

The locomotor activity of mice was monitored using the infrared motion detector. Activity counts accumulated over a 1 h period were measured for a total of 4 days in a 12 h light:12 h dark cycle (lights on at 8 am). **A:** Representative tracings of the locomotor activity from WT (top) and TG (bottom) mice. During the nighttime (8 pm to 8 am), the most mice showed characteristic biphasic patterns of locomotor activity, i.e., the 1st peak during the time period from 8 pm to 2 am, and the 2nd peak during the time period from 2 am to 8 am (arrows). **B:** The 1st and the 2nd peak activity during nighttime in WT (filled bar) and TG (open bar) mice. **C:** Total activity counts during daytime (8 am to 8 pm) in WT and TG mice. Data pooled for 4 days were shown. Data were analyzed from 7 WT mice and 8 TG mice at adult stages (9–16 wks of age). *** $p < 0.001$ as compared to WT by Student's two-tailed unpaired t-test. Error bar = SEM.

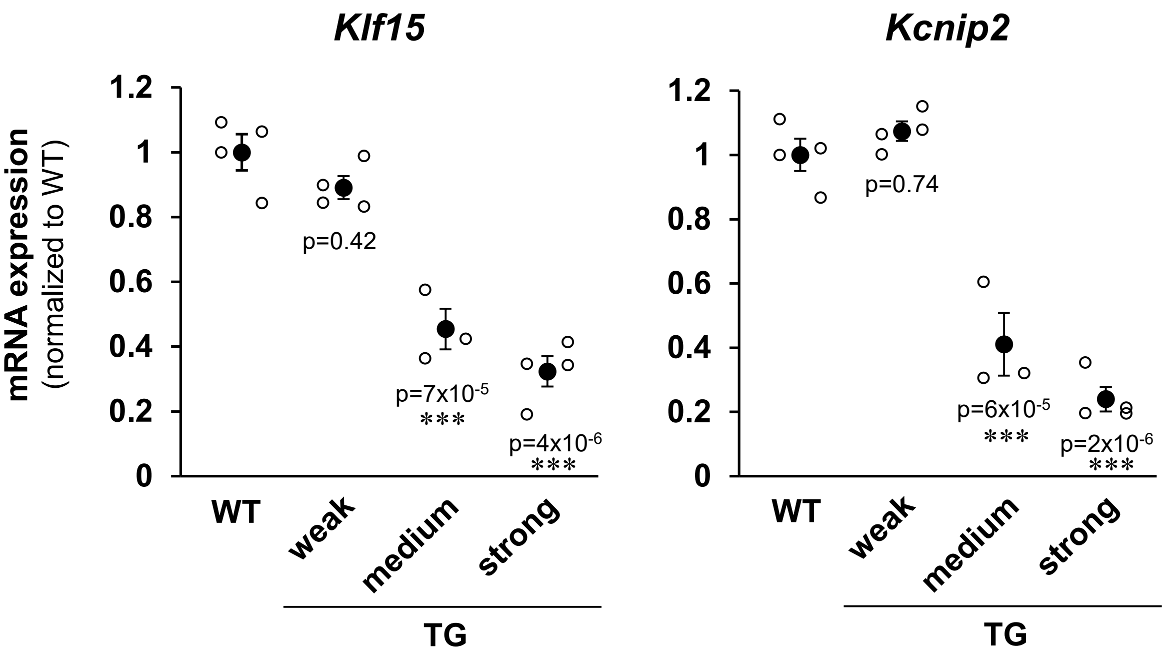

Figure S7. The extent of the repression of Klf15 and Kcnip2 were correlated with the expression level of Fam64a, related to Figure 5

qPCR analysis of *Klf15* and *Kcnip2* (KChIP2) in heart homogenates from WT and TG mice expressing various levels of Fam64a transgene (weak, medium, and strong) at 6 wks of age. Data were shown as normalized to WT. n = 3–4 mice per group. Error bar = SEM.. *** $p < 0.001$ as compared to WT by One-way ANOVA with Tukey's post hoc test.

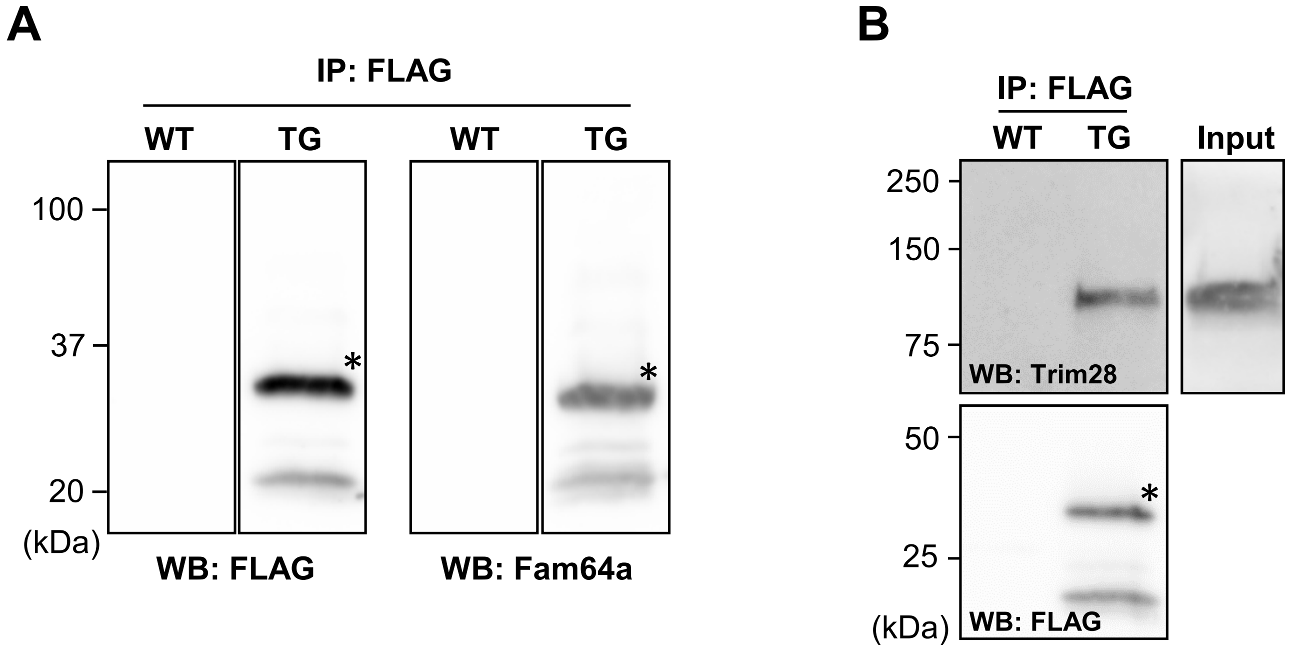

Figure S8. Comprehensive search for interacting partners of Fam64a, related to Figure 5.
A: Immunoprecipitation (IP) against FLAG peptide that was expressed as a C-terminal tag of overexpressing Fam64a protein in TG mice hearts, followed by western blotting (WB) using FLAG and Fam64a antibody, which correctly detected Fam64a-FLAG fusion protein (*) in TG, but not in WT mice heart lysates, validating the immunoprecipitation procedure. **B:** The same IP/WB procedure was applied for Trim28, indicating that Fam64a interacts with Trim28 in CMs.

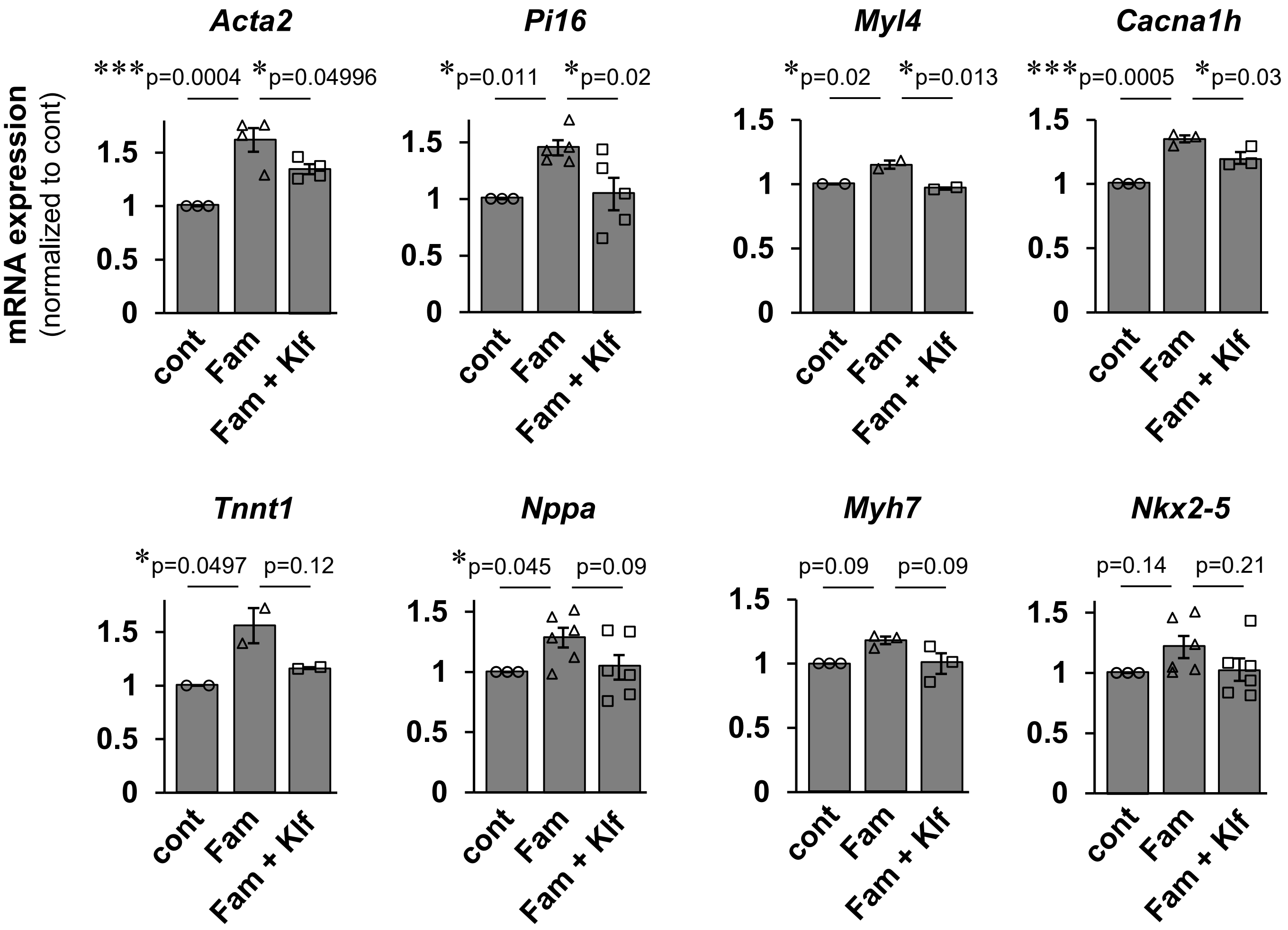

Figure S9. Fam64a-dependent induction of immature fetal genes was restored by concurrent expression of Klf15, related to Figure 5
Rescue experiments for Klf15 were performed using primary fetal CMs transduced with baculovirus expressing Fam64a alone (Fam) or both Fam64a and Klf15 (Fam + Klf) to examine whether forced expression of Klf15 restores the phenotypes induced by Fam64a overexpression. Data shown were qPCR analysis of immature fetal genes in each condition. Data were shown as normalized to controls. $n > 3$ independent experiments except for *Myl4* and *Tnnt1* evaluated with 2 independent experiments. * $p < 0.05$ and *** $p < 0.001$ between the indicated groups by One-way ANOVA with Tukey's post hoc test. Error bar = SEM. See legend for Figure 5G–J for details.

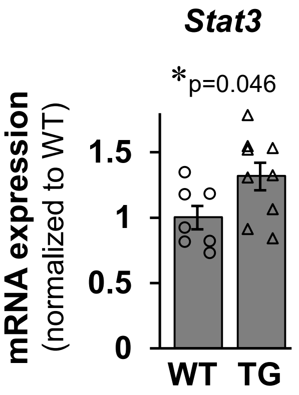

Figure S10. Stat3 mRNA was increased in Fam64a TG mice, related to STAR Methods
qPCR analysis of *Stat3* in WT and TG mice hearts. Data were shown as normalized to WT. n = 7–9 mice per group. * $P < 0.05$ as compared to WT by Student's two-tailed unpaired t-test. Error bar = SEM.

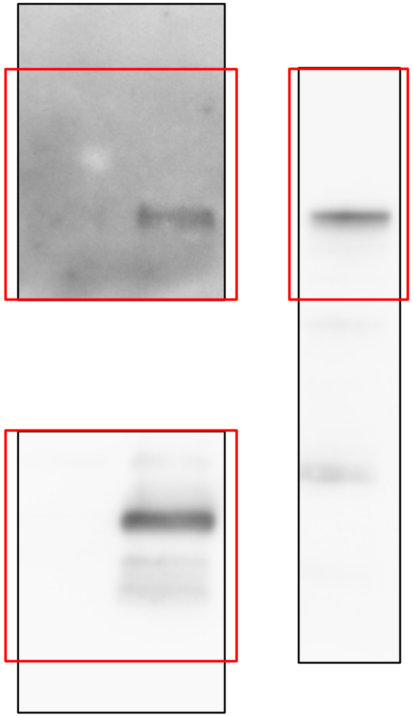

Figure 5B

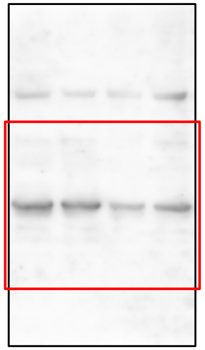

Figure 6I

Data S1. Full western blot images for Figure 5B and Figure 6I, related to STAR Methods

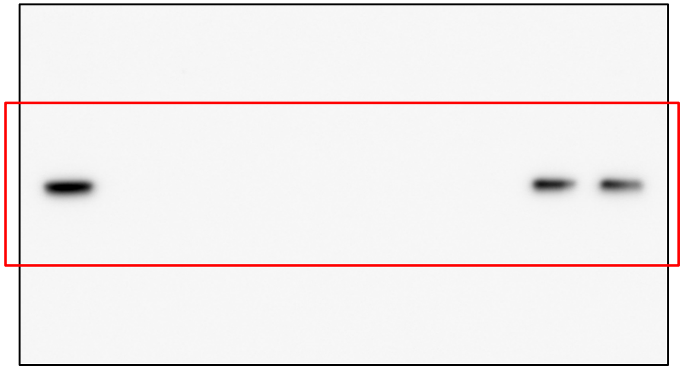

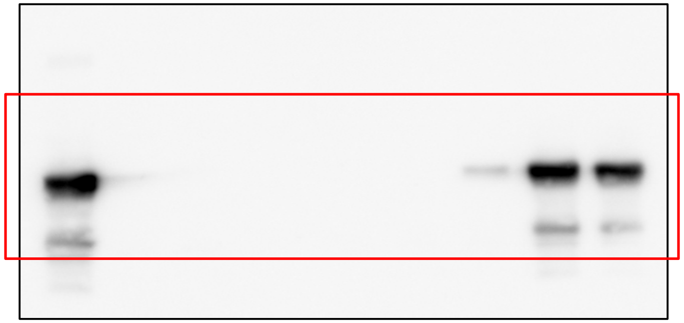

Figure S1C

Data S2. Full western blot images for Figure S1C, related to STAR Methods

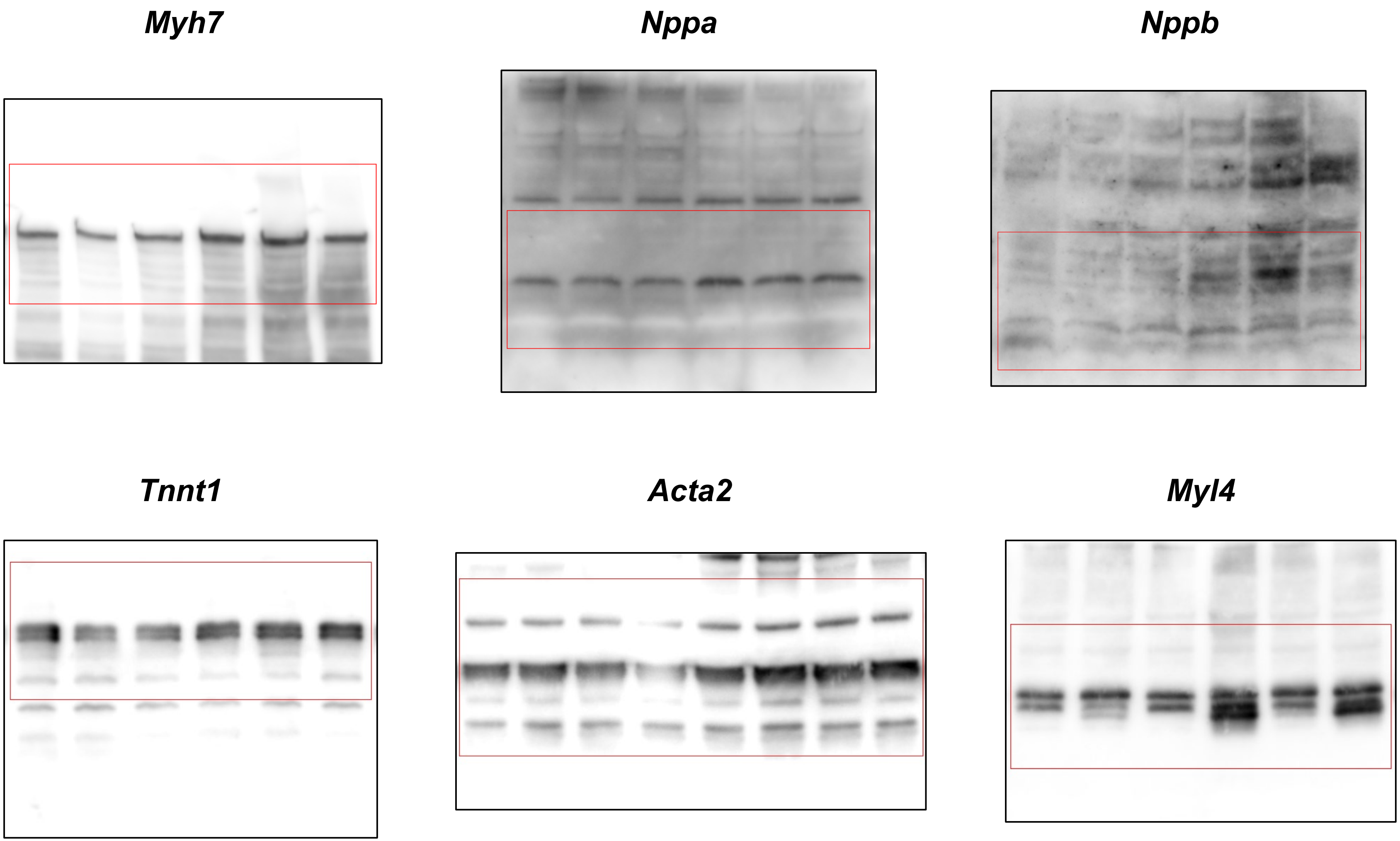

Figure S4

Data S3. Full western blot images for Figure S4, related to STAR Methods

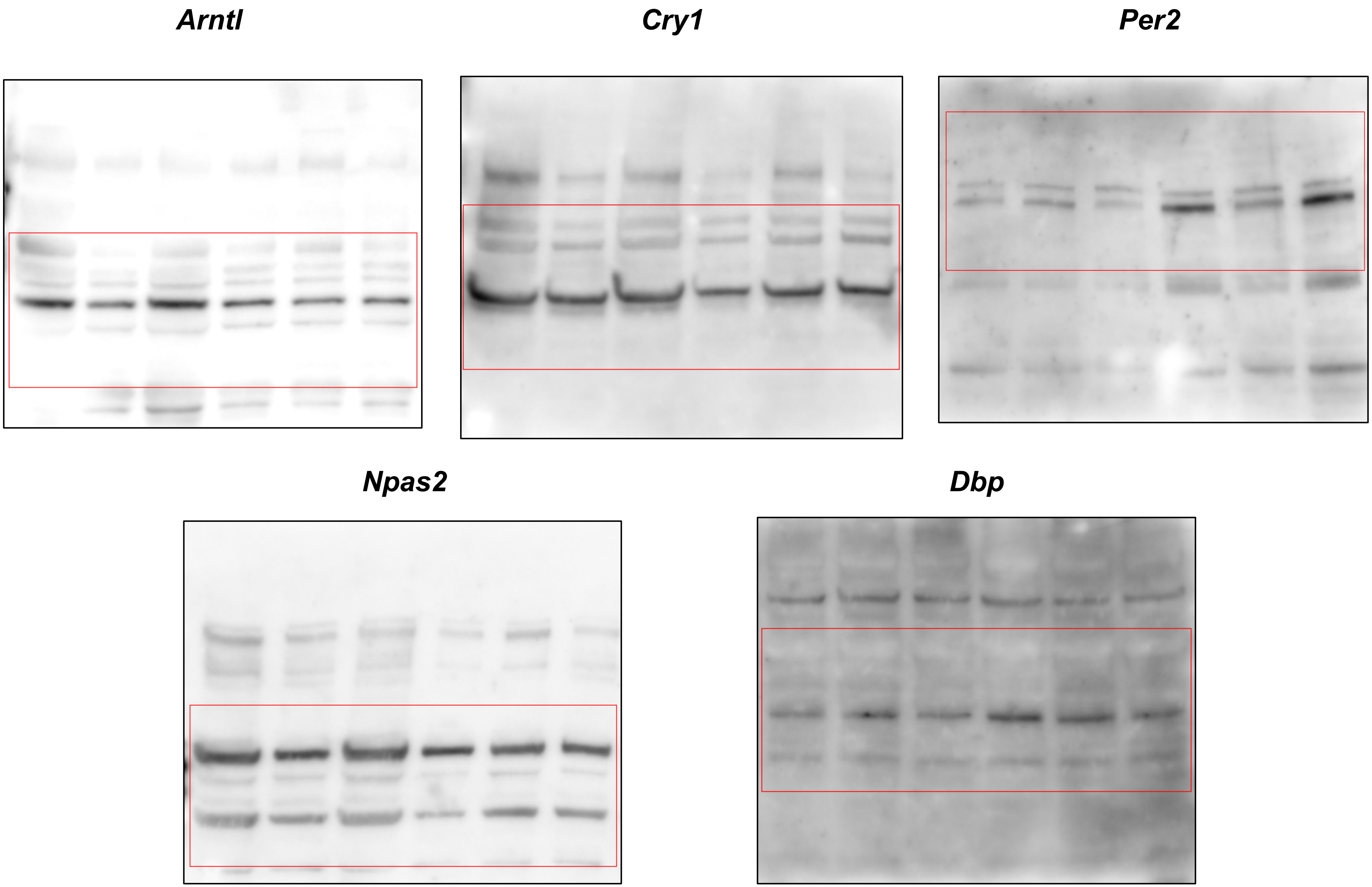

Figure S5

Data S4. Full western blot images for Figure S5, related to STAR Methods

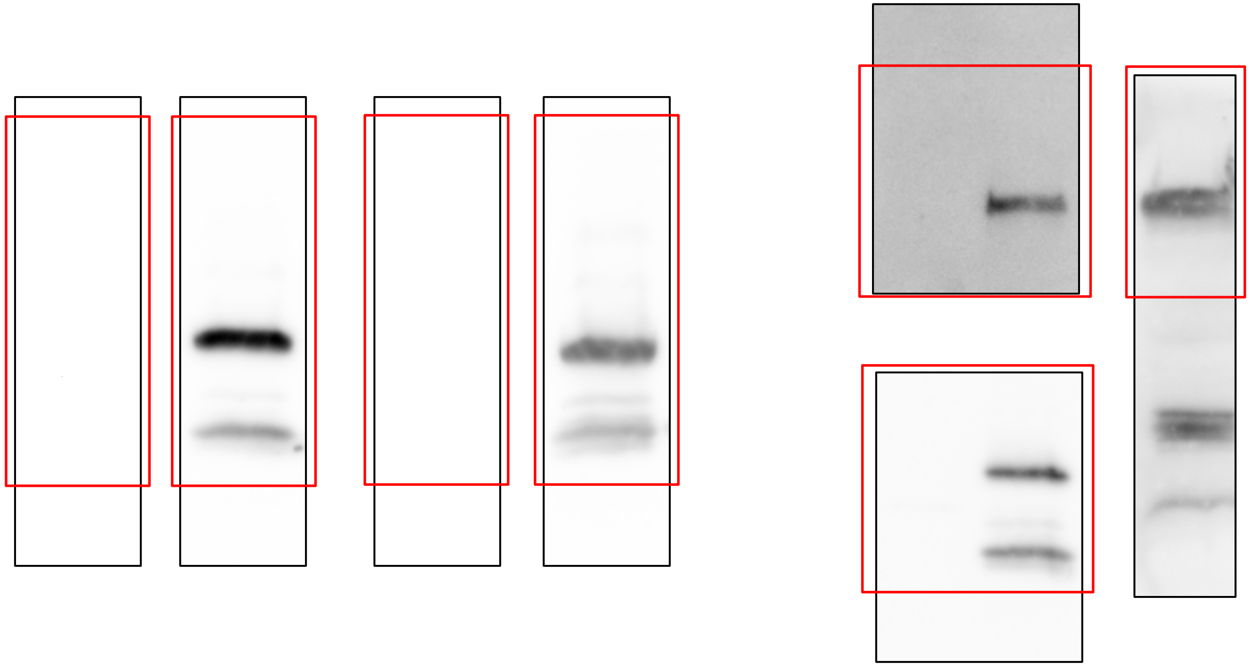

Figure S8

Data S5. Full western blot images for Figure S8, related to STAR Methods
